# AI-driven lithium-ion battery health prediction: a review of SOC, SOH and RUL estimation techniques

**DOI:** 10.3389/frai.2026.1858302

**Published:** 2026-10-14

**Authors:** G. Tamizharasi, G. K. Rajini

**Affiliations:** School of Electrical Engineering, Vellore Institute of Technology (VIT), Vellore, Tamil Nadu, India

**Keywords:** AI and DL, LiB monitoring, RUL, SOC, SOH

## Abstract

The need for precise lithium-ion battery (LiB) monitoring and prognostics has grown significantly with the increasing use of electric vehicles (EVs) and renewable energy system (RES) technologies. State of charge (SOC), state of health (SOH), and remaining useful life (RUL) are three critical performance metrics for ensuring battery safety, functional dependability, and effective energy utilization. Non-linear degradation patterns, operating uncertainties, and dynamic environmental fluctuations are frequently difficult for traditional electrochemical and empirical assessment methods to capture. As a result, recent research has focused on advanced data-driven techniques for comprehensive battery state estimation, notably those based on artificial intelligence (AI) and deep learning (DL). This review provides an overview of current methodologies for SOC, SOH, and RUL prediction, with a focus on modern DL architectures such as transformer networks, autoencoder-based latent modeling, neural basis expansion techniques, attention mechanisms, and hybrid optimization-driven frameworks. The article examines new advances in intelligent battery prognostics critically, emphasizing their potential for improved estimation precision, resilience, and flexibility in practical operating scenarios. In addition, it identifies future research directions such as enhanced optimization-assisted frameworks, hybrid physics-informed learning, and explainable artificial intelligence (XAI). This article intends to offer a comprehensive overview of modern battery prognostics approaches to aid in the development of dependable, efficient, and long-term battery management system (BMS) solutions for electric mobility and energy storage systems.

## 1 Introduction

One of the main causes of climate change and global warming is the sharp increase in greenhouse gas emissions. Environmental deterioration is still being exacerbated by emissions from fossil fuel-based transportation, industry, and traditional electricity generation. Reducing emissions from the energy and transportation sectors has become a major worldwide concern as countries work toward sustainable development goals and carbon neutrality (Jafari and Byun, 2022). The search for greener electrification technologies that can replace traditional combustion-based systems has intensified as a result of this shift. Strong technological and legislative solutions are necessary to address these environmental issues (Natella et al., 2022). Intelligent energy infrastructure, renewable power integration, and electric mobility are being promoted by governments and businesses worldwide (Wei et al., 2022a). Due to the sporadic nature of renewable resources like solar and wind, sophisticated energy storage technologies are essential for this shift. Demand-side management, grid balancing, and reliable energy distribution all depend on a dependable storage medium (Knox et al., 2024).

Battery-based energy storage systems (BESSs) have become the most viable and scalable storage technology. Batteries help microgrids increase system resilience, reduce dependence on fossil fuels, and enhance the utilization of RES (Karthick et al., 2025). EVs, smart grids, off-grid telecom systems, weather stations, portable gadgets, industrial backup systems, and renewable hybrid power plants all make extensive use of them. Their ability to store energy during periods of low demand and release it during periods of high demand makes them crucial to future sustainable infrastructure (Shu et al., 2022; Pramila et al., 2025). Table 1 provides a review of different battery types and their advantages and disadvantages.

**Table 1:** Review of battery technologies.

Battery type	Advantages	Limitations
Lead–acid	•Affordable and easy to manufacture •Low cost per kWh •Reduced self-discharge •High specific power •Improved performance over wide temperature range	•Low specific energy •Slow charging (14–16 h) •Needs charged storage •Limited deep-cycle life •Flooded-type transport limits •Environmental concerns
Nickel–Cadmium (Ni-Cd)	•Supports ultra-fast charging •Good load performance •Stored or discharged •Easy transport/storage •Good low-temperature operation	•Lower specific energy •Memory effect •Toxic cadmium disposal issue •High self-discharge •Low cell voltage (1.2 V)
Nickel–Metal Hydride (NiMH)	•Higher energy density than Ni–Cd •Environmentally safer •Moderate cycle life	•High self-discharge •Heat generation during fast charging
Sodium–Sulfur (NaS)	•Large energy density •Extended cycle life •Suitable for large-scale storage	•Large operating temperature •Safety concerns •Costly thermal insulation

Lithium-ion batteries (LiBs), in particular, are now extensively integrated into contemporary technology ecosystems (Guo and Shen, 2022). Because of their small size and lightweight design, LiBs are used in consumer electronics including wearables, computers, smartphones, and medical equipment (Singh et al., 2023). Because of their high power capacity and quick response time, they are the most popular energy source for EVs and hybrid cars in transportation (Li Y. et al., 2022). LiB packs provide frequency regulation, peak shaving, and backup operations in grid-scale storage, while they stabilize variable solar and wind outputs in renewable energy systems (Lu et al., 2023). Their real-world operation is defined by temperature fluctuations, dynamic load variations, and repeated cycles of charging and discharging. An important turning point in electrochemical energy storage was reached with the invention of LiB technology (Amir et al., 2022). Their high energy density, extended cycle life, high coulombic efficiency, and low self-discharge rate make them superior to conventional rechargeable batteries (Wang Y. et al., 2022). LiB production has quickly expanded into multi-terawatt-hour manufacturing capacity as EV adoption and renewable integration rise internationally. Their industrial importance has been reinforced by ongoing advancements in materials design, electrode chemistry, cell production, and pack-level BMS (Surya et al., 2022).

Reversible lithium-ion intercalation and deintercalation between the cathode and anode during charging and discharging control the operation of LiBs. While electrons flow through the external circuit during charging, lithium ions move from the cathode toward the anode through the electrolyte and separator (Qin et al., 2023). During discharge, the reverse electrochemical reaction generates useful electrical power, a process that, although it appears straightforward, actually involves complex, cross-scale interactions. From ion diffusion occurring within milliseconds to thermal dynamics unfolding across years of service life, the physical, chemical, thermal, and electrochemical phenomena are intricately intertwined. Many industries have changed as a result of the widespread use of LiBs. Large-scale renewable penetration is made possible by grid-connected battery farms, and their use in EVs directly contributes to transportation decarbonization and greenhouse gas mitigation (Qin et al., 2022). Over the next 10 years, it is anticipated that global battery production will surpass multi-terawatt-hour capacity, underscoring the strategic significance of LiBs in energy security, climate resilience, and transportation electrification. Nevertheless, such rapid adoption also makes lifetime optimization, safe operation, and precise health monitoring more important.

Despite their benefits, LiBs have several drawbacks that have an immediate impact on their lifetime and performance:

- **Capacity fade:** progressive decrease of charge storage capacity over a number of cycles.
- **SEI layer growth:** active lithium is continuously consumed during the development of the solid electrolyte interphase.
- **Lithium plating:** metallic lithium can be deposited during rapid charging or low-temperature operation.
- **Electrode micro-cracking:** structural fractures in active materials are caused by mechanical stress.
- **Increased internal resistance:** causes greater heat production and reduced power output.
- **Thermal stress:** aging processes are accelerated by high temperatures.
- **Thermal runaway:** an explosion or fire could result from localized overheating.
- **Cell inconsistency:** pack-level hotspots are produced by unequal heating and state mismatch.

Together, these degradation mechanisms shorten RUL, lower SOH, and make reliable BMS control difficult. Table 2 summarizes a review of battery state estimation methods.

**Table 2:** Evaluation of traditional battery state estimation methods.

Technique	Working Principle	Strength	Major Limitation
Capacity-based method	Uses charge/discharge capacity ratio	Direct SOH interpretation	Needs full cycle testing
Electrochemical impedance spectroscopy (EIS)	Uses impedance-frequency characteristics	Sensitive aging detection	Offline and costly
Equivalent circuit models (ECM)	Uses an RC equivalent electrical network	Fast implementation	Reduced physics fidelity
Electrochemical model	Uses diffusion and reaction equations	High physical accuracy	High computational burden
Kalman filter/observer	Recursive model correction using measurements	Good online tracking	Model mismatch sensitivity
Statistical regression	Learns signal-health relationship	Simple and fast	Poor adaptability

### 1.1 Research gap

Even though conventional battery state estimation methods have made significant strides, as Table 2 summarizes, there are still a number of unmet research gaps. Under the non-linear, multifactorial, and time-varying operating conditions typical of contemporary LiBs, conventional techniques like capacity-based estimation, EIS, ECM, and Kalman filter observers often struggle to capture hidden degradation dynamics. These constraints make it difficult to accurately estimate SOH and RUL, particularly when dealing with different chemistries and dynamic load profiles.

Artificial intelligence (AI)-driven prognostics and diagnostics have emerged as viable solutions to these drawbacks. Without the need for explicit physical simplifications, machine learning (ML) and deep learning (DL) frameworks can understand intricate connections between voltage, current, temperature, impedance, and cycle behavior (Che et al., 2023). Additionally, to improve reliability across a range of chemistries and operating conditions, hybrid physics-informed AI models combine data-driven intelligence with electrochemical understanding.

As a result, the shift to AI-enabled BMS is crucial for enhancing safety, prolonging lifespan, lowering maintenance costs, and supporting the world's clean energy transition (Yao Q.et al., 2022). For next-generation EVs, smart grids, and large-scale energy storage systems, this research gap emphasizes the need for unified frameworks that combine physical interpretability with data-driven adaptability.

## 2 Definitions

### 2.1 Battery dataset analysis

Three critical parameters are estimated for accurate battery status monitoring: SOC, SOH, and RUL. Together, these factors characterize the battery's current energy availability, deterioration state, and potential future use, respectively. To enable accurate forecasting and energy management, these indicators are derived from voltage, current, temperature, and capacity readings in battery dataset analysis (Peng et al., 2022). Figure 1 shows the definitions of state of charge, state of health, and remaining useful life.

**Figure 1:**
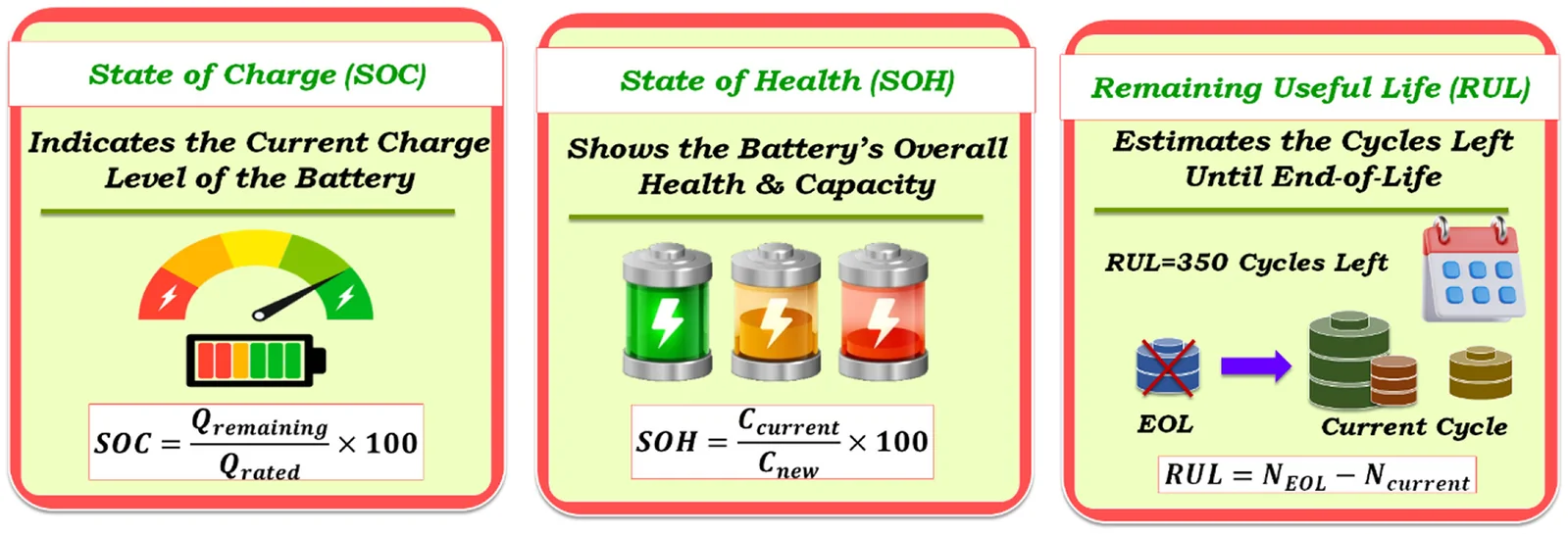
Definitions of battery state of charge (SOC), state of health (SOH), and remaining useful life (RUL).

### 2.2 State of charge (SOC)

SOC shows the battery's current charge in relation to its rated capacity. It indicates the proportion of energy that is still available for usage right now and changes dynamically depending on charging and discharging circumstances. It represents the battery's current operational state; SOC is one of the most crucial real-time battery monitoring metrics. SOC is stated mathematically as in Equation 1:


$$\begin{array}{l} SOC=\frac{Q_{remaining}}{Q_{rated}}\times 100 \end{array}$$ (1)

where *Q*_*remaining*_ indicates the battery's remaining charge and *Q*_*rated*_ reflects the nominal rated capacity of a fully charged battery.

A battery with more available charge is indicated by a higher SOC value, while one that is nearing depletion is indicated by a lower SOC value. For BMS, EVs, and RES, where precise charge tracking ensures safe and effective operation, SOC estimation is crucial (Karthick, 2025).

### 2.3 State of health (SOH)

SOH compares a battery's current status to its initial healthy state. The battery's maximum usable capacity gradually declines as it ages and experiences repeated charge-discharge cycles, which lowers SOH. Internal resistance growth or capacity decline is frequently used to describe battery SOH. The battery dataset's degradation pattern is directly captured by capacity-based SOH.

The definition of SOH is provided in Equation 2:


$$\begin{array}{l} SOH=\frac{C_{current}}{C_{new}}\times 100 \end{array}$$ (2)

where *C*_*current*_ is the old battery's maximum capacity in the current cycle and *C*_*new*_ is the new battery's rated capacity.

The SOH value steadily drops from 100% as aging advances toward the end-of-life (EOL) threshold, which is typically thought to be around 80% capacity retention. As a result, SOH is an essential health indicator for lifespan forecasting and degradation modeling (Gomathi, 2025).

### 2.4 Remaining Useful Life (RUL)

The estimated number of cycles or operating times left until a battery reaches its EOL condition is represented by its RUL. It offers a future-focused forecast of battery usage, in contrast to SOC and SOH, which reflect the current battery condition.

The battery's historical deterioration trajectory, which includes capacity loss, resistance increase, temperature behavior, and cycling circumstances, is used to determine RUL. The remaining cycle count needed for the current SOH to drop to the EOL threshold is referred to in this study as RUL. It is expressed mathematically as in Equation 3:


$$\begin{array}{l} RUL=N_{EOL}-N_{current} \end{array}$$ (3)

where *N*_*current*_ is the current cycle number and *N*_*EOL*_ is the cycle number at which the battery reaches the end-of-life condition (Jafari and Byun, 2023).

## 3 Review of SOC estimation

### 3.1 Gated recurrent unit (GRU)-based encoder–decoder network

For battery SOC estimation, the GRU-based encoder–decoder network presented in (Yang et al., 2022) is a powerful DL framework that models non-linear temporal relationships in battery signals. The architecture is intended to address the sequence-to-sequence learning challenge, which involves mapping past measurements like temperature, voltage, and current to the current SoC value. An encoder, a latent state vector, and a decoder make up the framework. Figure 2 denotes the GRU based SOC estimation.

**Figure 2:**
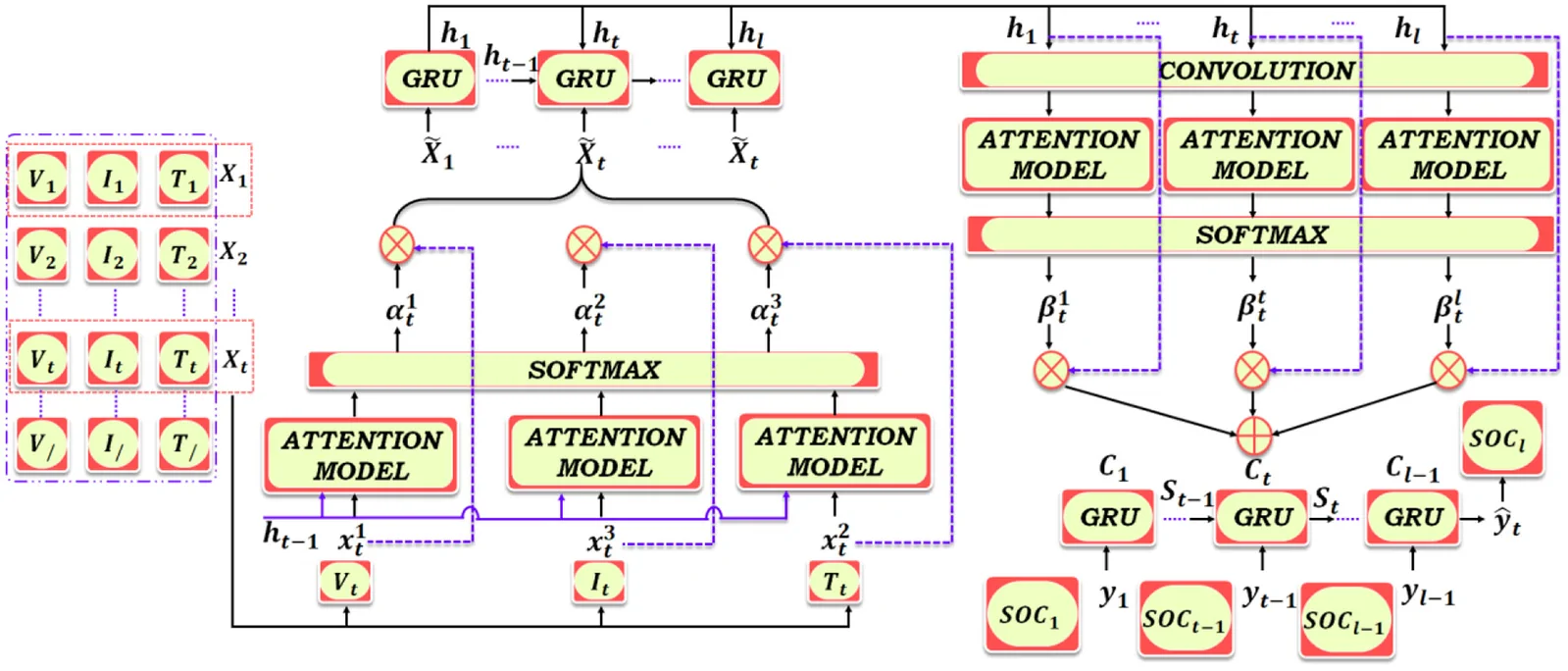
Gated recurrent unit (GRU) dual-attention-based state-of-charge (SOC) estimation.

The multivariate input sequence *X*_*t*_ = [*V*_*t*_, *I*_*t*_, *T*_*t*_] is first received by the encoder, which transforms it into hidden representations that capture temporal battery dynamics. At time *t*, the GRU hidden state is updated through the following mechanism as in Equations 4–7:


$$\begin{array}{lll} z_{t} & = & \sigma \left(W_{z}•\left[h_{t-1},X_{t}\right]\right) \end{array}$$ (4)

$$\begin{array}{lll} r_{t} & = & \sigma \left(W_{r}•\left[h_{t-1},X_{t}\right]\right) \end{array}$$ (5)

$$\begin{array}{lll} {\tilde{h}}_{t} & = & tanh\left(W_{\tilde{h}}•\left[r_{t}⊙h_{t-1},X_{t}\right]\right) \end{array}$$ (6)

$$\begin{array}{lll} h_{t} & = & \left(1-z_{t}\right)⊙h_{t-1}+z_{t}⊙{\tilde{h}}_{t} \end{array}$$ (7)

Here, *z*_*t*_ and *r*_*t*_ stand for the update and reset gates, respectively, which control the flow of information and determine whether the previous state is retained or reset. The previous hidden state is represented by *h*_*t*−1_. This approach emphasizes how a GRU differs from a typical RNN by minimizing gradient vanishing and specifically regulating long-term dependence.

The model incorporates a dual-stage attention technique to improve prediction accuracy further. This GRU encoder–decoder with attention greatly improves prediction accuracy, temporal feature extraction, and SOC estimate robustness, making it ideal for sophisticated BMS.

### 3.2 Extended Kalman filter (EKF)

A LiB's SOC is estimated using an EKF, which performs well when estimating SOC due to its ability to predict the non-linear dynamics of the battery while having low computational complexity and fast convergence times. In a first-order RC battery model, SOC and polarization voltage are state variables, and the terminal voltage is an observation variable. The relationship between the open-circuit voltage (OCV) and SOC is non-linear; therefore, the EKF uses a first-order Taylor series to linearize the non-linear state-space model at each instant in time during the sampling period. The generalized non-linear state-space model expressed in Equations 8, 9 represents the EKF battery model.


$$\begin{array}{l} x_{k}=f\left(x_{k-1},u_{k-1}\right)+w_{k-1} \end{array}$$ (8)

$$\begin{array}{l} y_{k}=h\left(x_{k},u_{k}\right)+v_{k} \end{array}$$ (9)

Here, *x*_*k*_ is the state vector and polarization voltage, *u*_*k*_ is the input current and *y*_*k*_ is the measured terminal voltage. *w*_*k*_ is independent, zero-mean Gaussian process noise, and *v*_*k*_ is independent, zero-mean Gaussian measurement noise. By using this non-linear formulation of the state-space model, EKF recursively predicts and corrects the estimated states of SOC and polarization voltage by performing *a priori* estimation, Kalman gain calculation, and updating states after measurement to provide accurate real-time estimates of SOC with increased stability and robustness (Wang Z. et al., 2022).

### 3.3 Time-delayed recurrent neural network (TD-RNN)

TD-RNN is represented in Figure 3 and captures the dynamic non-linear temporal behavior of LiBs for battery terminal voltage modeling and SOC estimation. This method uses the input vector *x*_*i, k*_ to represent the battery's SOC and current profile, where *i* stands for the input variable and *k* for the discrete time step. By utilizing both the present input data and the delayed hidden-state feedback from earlier time steps, TD-RNN enables the model to acquire electrochemical hysteresis features and battery memory effects.

**Figure 3:**
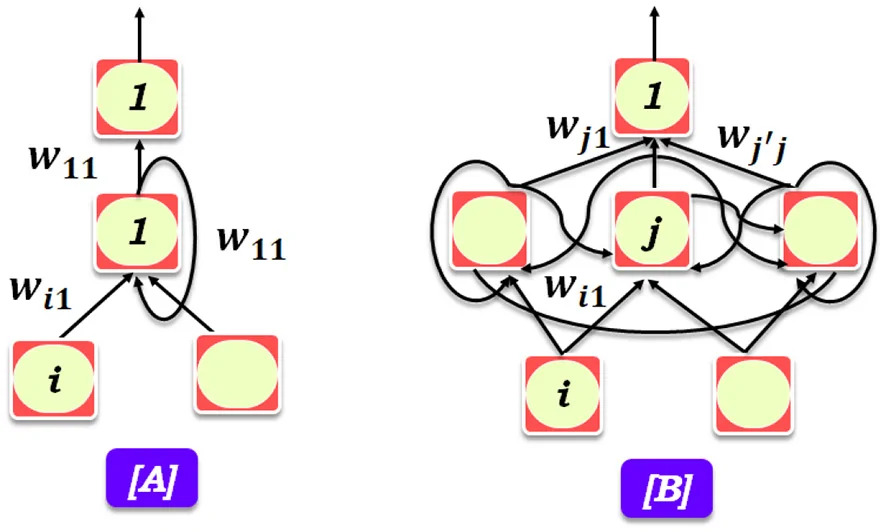
Schematic diagram of a time-delayed recurrent neural network (TD-RNN). **(A)** Single neuron. **(B)** Multi-neuron.

The hidden neuron input at time step *k* in the TD-RNN architecture, which has one hidden neuron and one time-delay step, is written as in Equation 10:


$$\begin{array}{l} z_{1,k}=\underset{i}{\sum }w_{i}x_{i,k}+w_{r}y_{1,k-1}+b_{1} \end{array}$$ (10)

The input weights are represented by *w*_*i*_, the recurrent feedback weight is represented by *w*_*r*_, the prior hidden-state output is represented by *y*_1, *k*−1_ and *b*_1_ is the bias. A hyperbolic tangent sigmoid activation function is used to produce non-linear hidden-state output. A linear output layer is then used to calculate the anticipated terminal voltage.

The trained TD-RNN precisely maps the time-series relationship between SOC, current, and terminal voltage since terminal voltage and SOC have a significant correlation. This allows for accurate SOC prediction (Xi et al., 2022).

### 3.4 Genetic algorithm (GA)-GRU

The GA–GRU model in Figure 4 is an efficient data-driven method for battery SOC estimation and is especially well-suited for non-linear and long-term temporal battery datasets. The GRU network is intended to resolve gradient vanishing and long-term dependency issues that arise in traditional RNNs. GRU employs two gating mechanisms: reset and update gates; this lowers computational complexity and speeds up convergence. The time-series battery measurements comprise an input sequence for battery SOC calculation. These measurements are represented as *x*_*t*_ = [*V*_*t*_, *I*_*t*_, *T*_*t*_].

**Figure 4:**
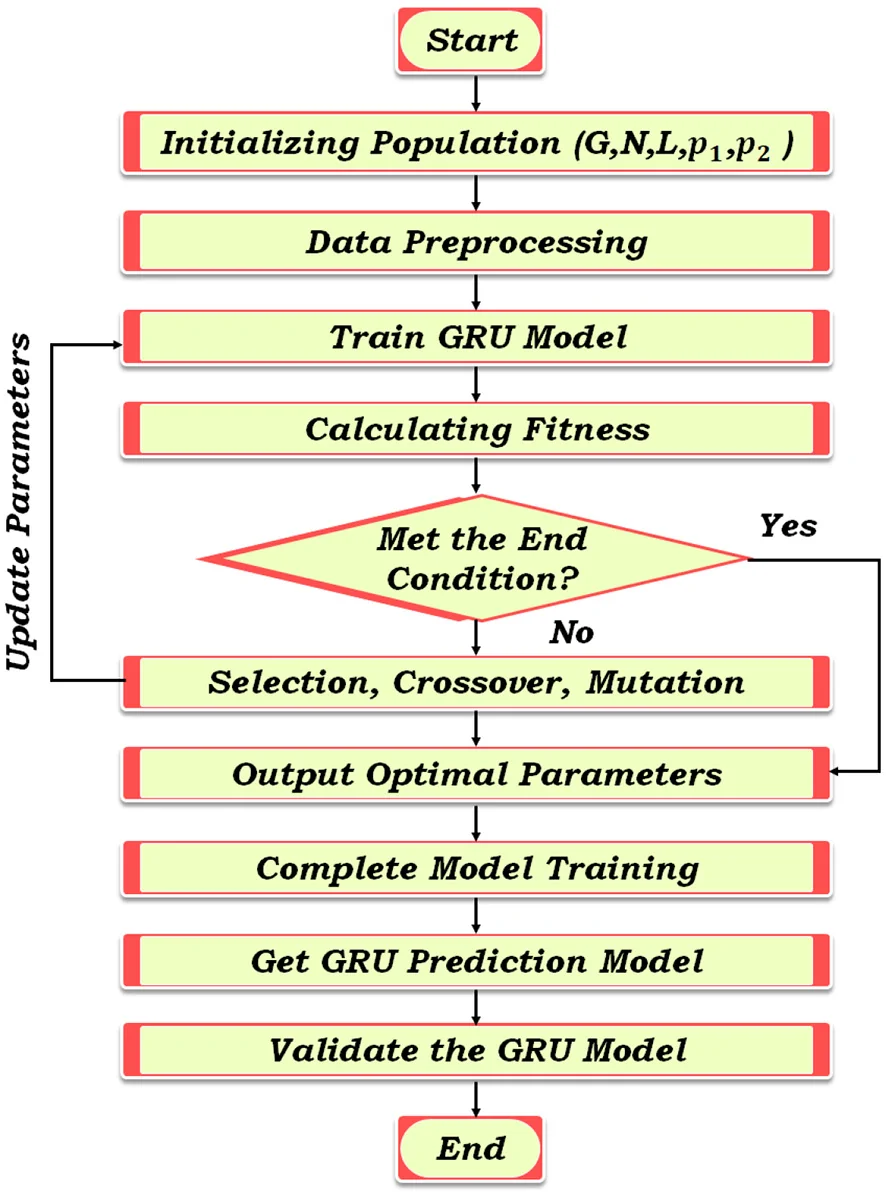
Genetic algorithm–gated recurrent unit (GA-GRU) implementation process.

The sigmoid and hyperbolic tangent functions are the activation functions employed as in Equation 11:


$$\begin{array}{l} \sigma \left(x\right)=\frac{1}{1+e^{-x}} \end{array}$$ (11)

The GRU output layer predicts the SOC value *y*_*t*_, where the training dataset is written as {(*x*_1_, *y*_1_), (*x*_2_, *y*_2_), ..., (*x*_*n*_, *y*_*n*_) }.

To further increase prediction accuracy, a GA is integrated for hyperparameter tuning of the GRU model. By using selection, crossover, and mutation procedures, the ideal design characteristics are determined; the GA simulates biological evolution. In this work (Chen J. et al., 2022), the search ranges are chosen between 1–3 layers and 32–256 neurons, and the GA optimizes the number of GRU layers, neurons, fully connected layers, and their neuron sizes. The mean squared error (MSE) of SOC prediction is selected as the fitness function:


$$\begin{array}{l} MSE=\frac{1}{n}\overset{n}{\underset{i=1}{\sum }}{\left({ŷ}_{i}-y_{i}\right)}^{2} \end{array}$$ (12)

where ŷ_*i*_ and *y*_*i*_ stand for current and anticipated SOC values, respectively. Random population initialization is the first step in the GA process, after which each GRU configuration's fitness is assessed. Until the stopping criterion is met, high-fitness chromosomes are chosen, crossed, and modified repeatedly. The final GA–GRU SOC estimator, which exhibits better accuracy, shorter training times, and increased robustness under dynamic battery operating conditions, is then built using the optimized parameters. To highlight how health degradation analysis enhances charge estimation in thorough battery diagnostics, this section ends with a transition to the next section on SOH estimation techniques.

## 4 Review of SOH estimation approaches

### 4.1 Physics-informed neural network

PINNs increase model generalizability, decrease retraining effort, and improve prediction reliability by incorporating physical principles into the neural network learning process. In Figure 5, an additional physics-based loss function is used in battery health estimation applications to incorporate physical restrictions, such as the monotonic relationship between incremental capacity (IC) characteristics and SOH, into the training framework. This improves estimation accuracy across various battery datasets by allowing the network to simultaneously learn deterioration physics and data features.

**Figure 5:**
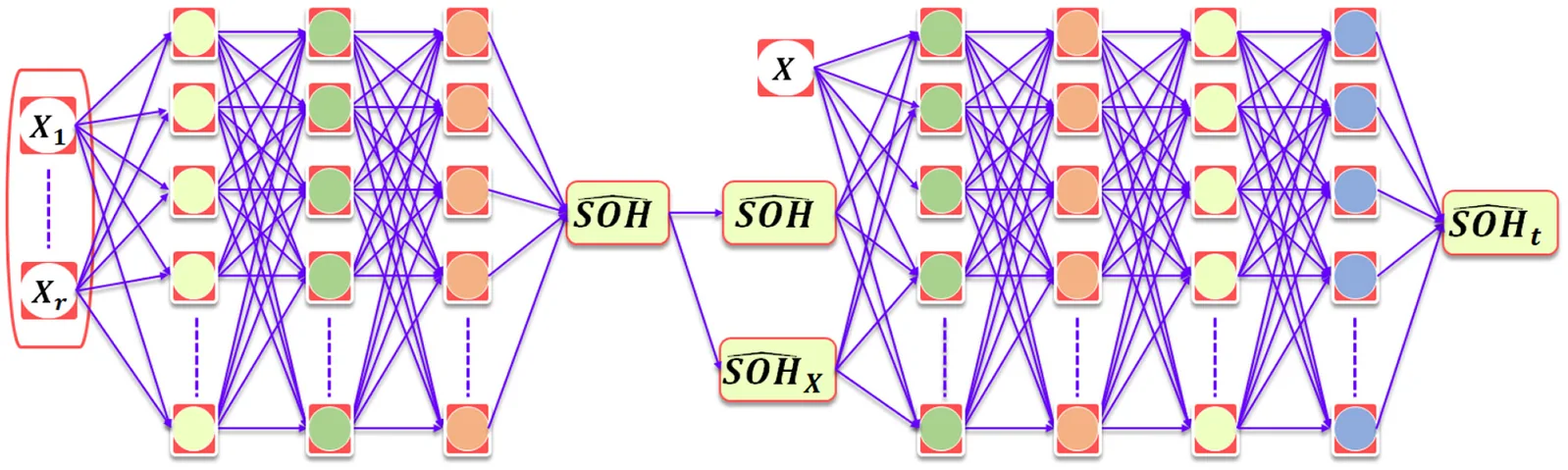
Physics-informed neural network (PINN) model.

The following expression represents the model's monotonicity constraint in Equation 13:


$$\begin{array}{l} L_{mono}=\frac{1}{N}\overset{n}{\underset{i=1}{\sum }}ReLU\left(\frac{\partial SOH}{\partial X_{IC}}\right) \end{array}$$ (13)

The partial derivative of projected SOH with respect to the IC feature *X*_*IC*_ is indicated by $\left(\frac{\partial SOH}{\partial X_{IC}}\right)$. The PINN architecture ensures physically consistent learning and enhances robustness under various battery operating situations by incorporating such physical knowledge into the optimization process (Deng et al., 2025).

### 4.2 Graph convolutional networks (GCNs)

GCNs, visualized in Figure 6, simulate the inter-cycle dependency and degradation correlation among battery charge/discharge cycles for battery SOH estimation. Each battery cycle in this method is represented as a node in a graph *G*(*V, E*), where *V* stands for the set of cycles and *E* for correlations between them. Each cycle's voltage segment is used as a node feature to create a feature matrix *X*. The adjacency matrix is created by utilizing discharge voltage profile similarity, which is typically measured using the Pearson correlation coefficient. GCNs learn both long-term degradation patterns and local cycle behavior due to graph representation.

**Figure 6:**
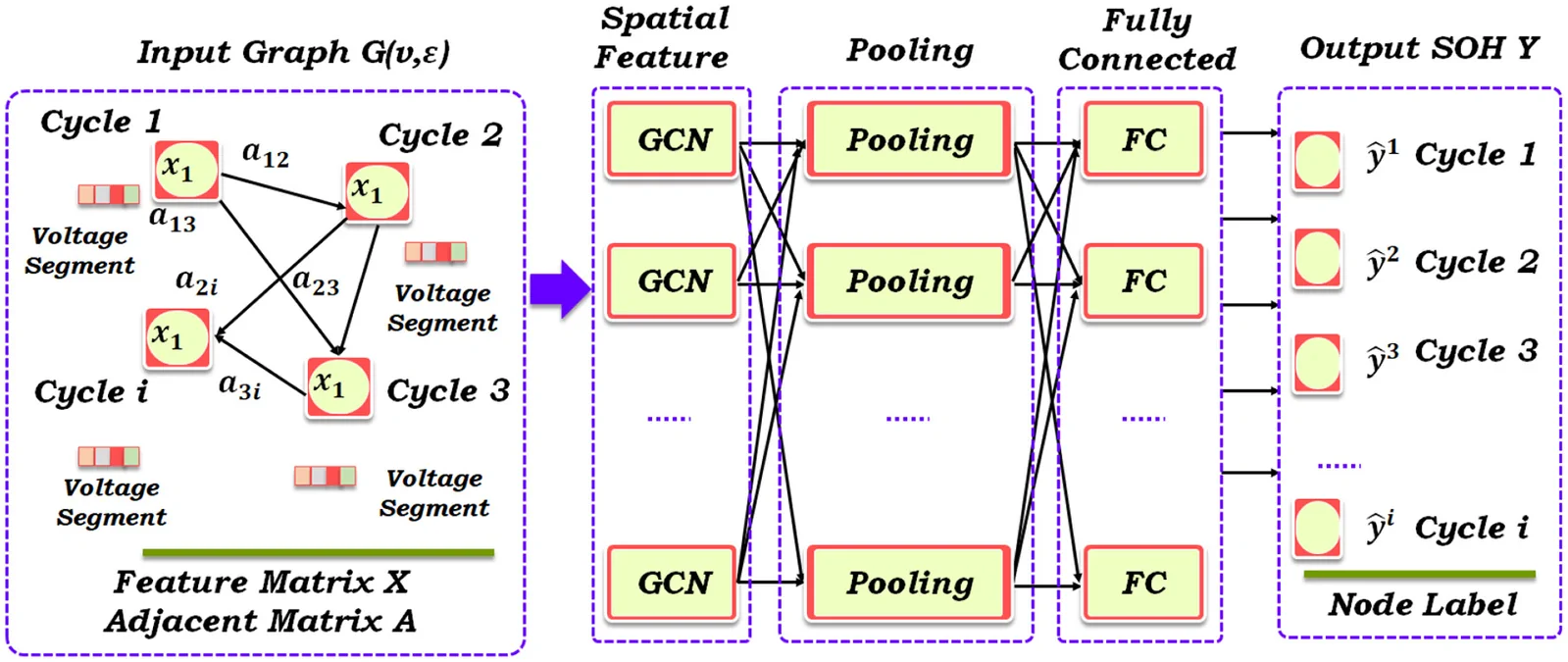
Graph convolutional network (GCN)-based SOH estimation model.

The graph convolution process updates node features layer by layer by combining neighborhood data as in Equation 14:


$$\begin{array}{l} H^{\left(l+1\right)}=\sigma \left({\hat{D}}^{-1/2}A{\hat{D}}^{-1/2}H^{\left(l\right)}W^{\left(l\right)}\right) \end{array}$$ (14)

where *W*^(*l*)^ is the trainable weight matrix of *l*^*th*^ layer, $\hat{D}$ is the diagonal degree matrix, and the non-linear activation function is represented by σ(·). The network generates SOH predictions for every cycle node via several GCN layers, pooling, and fully connected layers.

Voltage segment selection is a crucial preprocessing step in which the matrix profile (MP) approach is used to extract the most informative discharge voltage region. The newly observed cycle is added to the base graph during live SOH estimation, and the trained GCN model predicts its SOH as follows provided in Equation 15:


$$\begin{array}{l} {ŷ}_{\gamma }=F_{GCN}\left(X_{n+1}^{\gamma },A_{n+1}^{\gamma }\right) \end{array}$$ (15)

Therefore, cycle-to-cycle degradation propagation, voltage pattern evolution, and non-linear health decay are all well captured by GCN-based SOH modeling, which makes it ideal for precise online lithium-ion battery SOH estimation (Zhou et al., 2024).

### 4.3 Regression vision transformer (*R*-ViT)-based deep neural network

The ViT-based regression network introduced in (Chen L. et al. 2023) is an effective DL method for battery SOH prediction (Figure 7). By utilizing the powerful capacity of self-attention to simulate long-range dependencies in temporal battery data, this approach expands the transformer encoder architecture for battery degradation prediction. For extended battery cycle datasets, the transformer's simultaneous processing of all time steps allows for parallel computation and increased learning efficiency.

**Figure 7:**
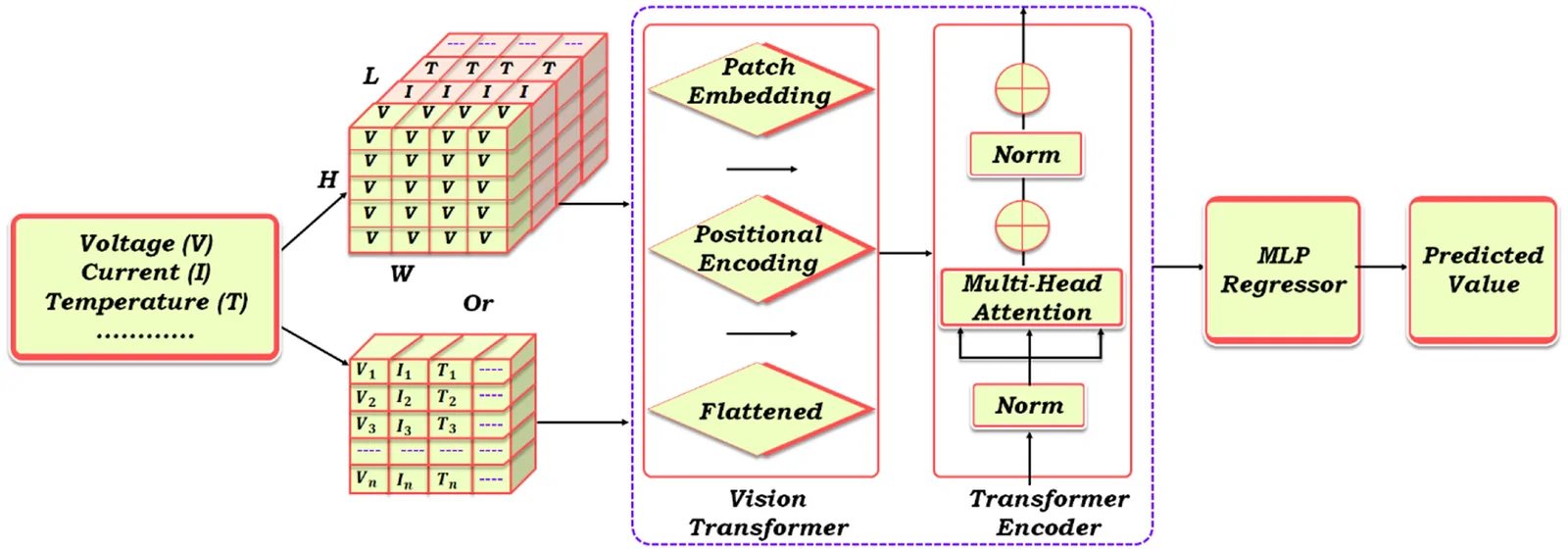
Regression vision transformer (*R*-ViT) architecture.

In the self-attention mechanism, each embedded input vector *a*_*i*_ is converted into a value (*v*_*i*_ = *W*_*v*_*a*_*i*_), a key (*k*_*i*_ = *W*_*k*_*a*_*i*_) and query vectors (*q*_*i*_ = *W*_*q*_*a*_*i*_). The attention score between features is $\alpha_{i}=\frac{q_{i}\cdot k_{i}}{\sqrt{d_{k}}}$, and the normalized softmax attention is $\alpha_{i}=\;\frac{\exp\left(\alpha_{i}\right)}{\underset{j}{\sum }\exp\left(\alpha_{j}\right)}$.

The traditional transformer is modified into an *R*-ViT architecture using just the encoder section in order to increase SOH prediction capacity. First, a three-dimensional tensor is created from the one-dimensional battery cycling features, which are then divided into patches. The *R*-ViT model concurrently captures temporal linkages among cycles and correlations among numerous health parameters.

### 4.4 Artificial Bee colony-based Bi-directional GRU (ABC-Bi-GRU)

The ABC-BiGRU model is illustrated in Figure 8 and learns the non-linear temporal dynamics of lithium-ion batteries among DL techniques for SOH estimation. Recurrent neural architectures are ideal for extracting degradation and charge-related characteristics that comprise a time-dependent sequential dataset with significant long-term correlations. While conventional RNNs are capable of modeling temporal sequences, their capacity to represent long-range electrochemical dynamics is diminished by vanishing- and exploding-gradient issues.

**Figure 8:**
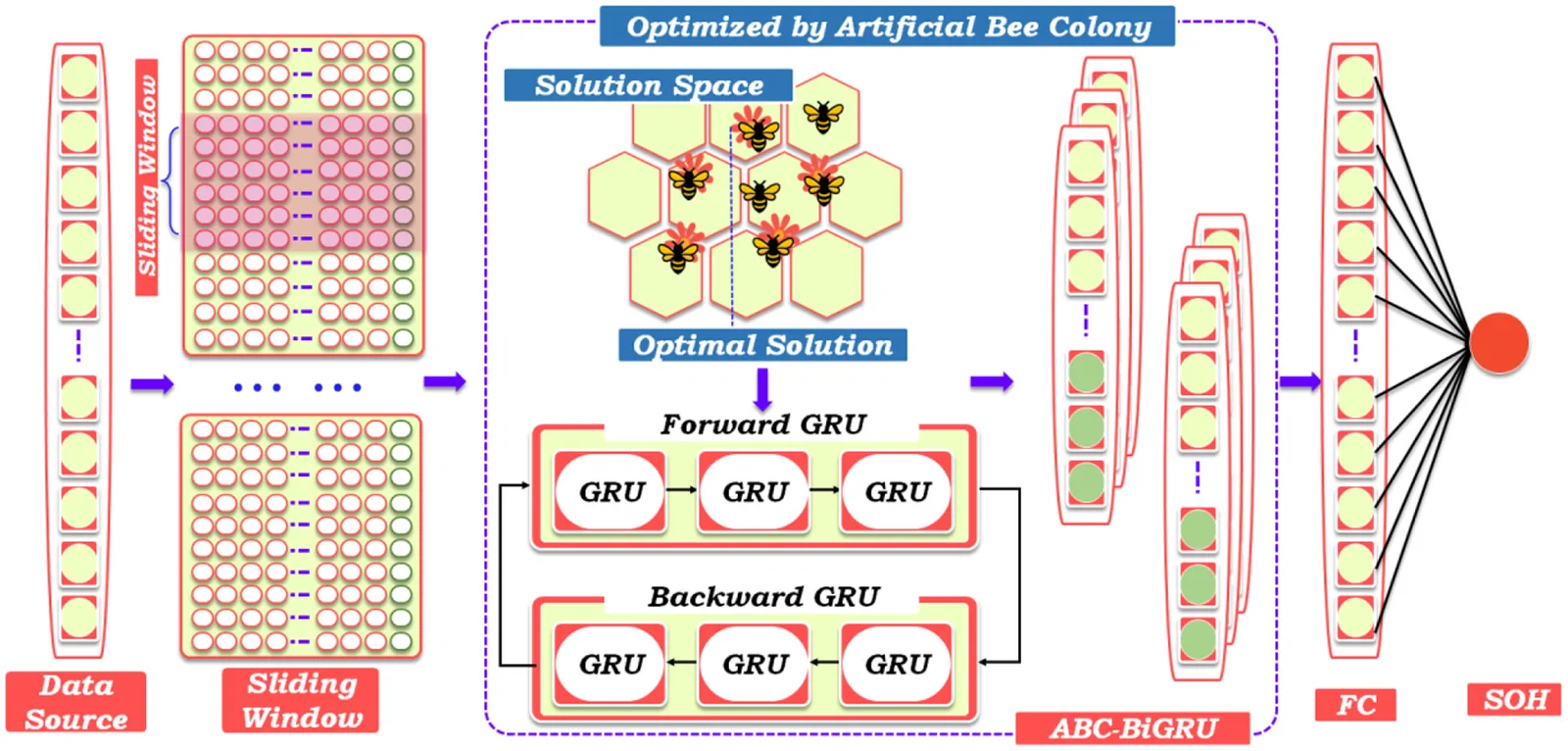
Artificial bee colony (ABC)-based bidirectional gated recurrent unit (Bi-GRU) model.

The GRU unit uses update and reset gates to effectively control memory flow in order to overcome this restriction. The Bi-GRU architecture simultaneously extracts historical charging trends and future discharge behavior by processing the battery sequence in both forward and backward directions, improving SOC prediction accuracy. As SOH trajectories are impacted by past current profiles, current terminal voltage, and future transient responses, this bidirectional learning is very advantageous. The ABC technique is used to adjust important Bi-GRU hyperparameters such as the number of hidden neurons, learning rate, and batch size.

$x_{ij}=x_{j}^{\min}+rand\left(0,1\right)\left(x_{j}^{\max}-x_{j}^{\min}\right)$ is the initialization of candidate solutions, whereas the updated solution is found as $x_{ij}^{new}=x_{ij}+\phi_{ij}\left(x_{ij}-x_{kj}\right)$. The ABC-Bi-GRU model greatly increases SOC estimation accuracy, convergence speed, and robustness by integrating bidirectional temporal learning of Bi-GRU with the global hyperparameter-search capability of ABC. This makes it ideal for sophisticated BMS applications in EVs and energy storage systems (ESSs) (Chen D. et al., 2022).

## 5 Review of RUL estimation approaches

### 5.1 Gaussian process regression (GPR)

Gaussian process regression (GPR) is a probabilistic, non-parametric ML technique based on Bayesian theory. In GPR (Figure 9), a Gaussian process prior is used to model the relationship between input and output variables, and a mean function and a kernel (covariance) function are used to represent the prediction. The model starts with an assumed prior distribution, updates it with the training observations, and then uses the posterior distribution to forecast data samples that have not yet been seen. GPR is ideal for sophisticated regression applications because it quantifies prediction uncertainty and model non-linear interactions.

**Figure 9:**
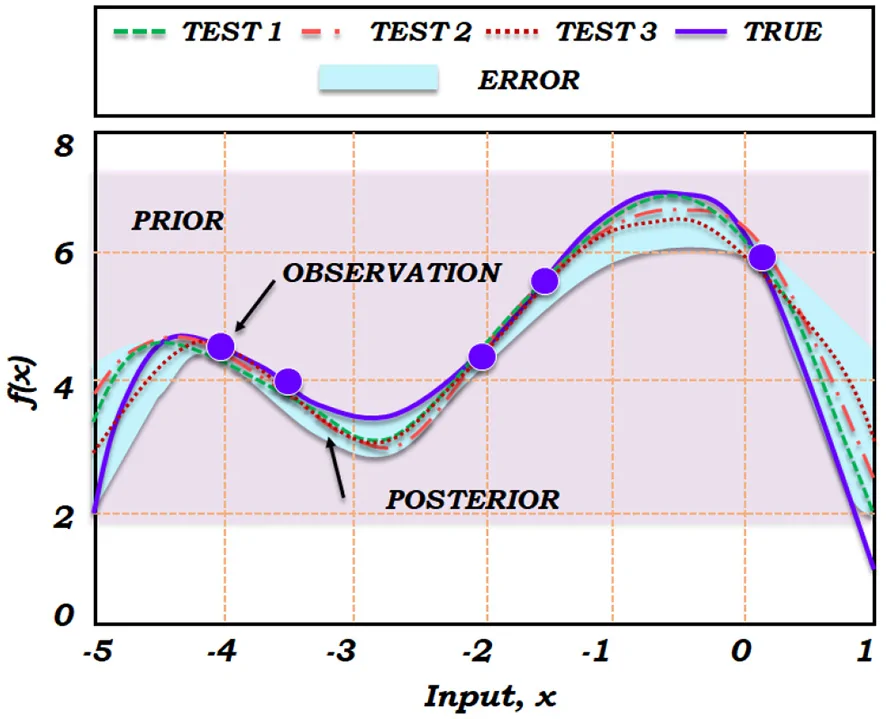
Gaussian process regression (GPR) algorithm.

The mathematical expression for the GPR model is in Equation 16:


$$\begin{array}{l} y=f\left(x\right)+\varepsilon \end{array}$$ (16)

where ε is an independently distributed Gaussian noise term, *f*(*x*) is the latent regression function, and *y* is the observed output. As a result, GPR delivers accurate prediction estimation with uncertainty characterization and efficiently learns the underlying functional behavior from observed data (Fu et al., 2025).

### 5.2 Denoising transformer-based neural network (DTNN)

DTNN, presented in Figure 10, is a sophisticated data-driven DL technique for precise RUL assessment of LiBs that utilizes both local signal features and long-term temporal correlations in historical battery data. In order to increase convergence and numerical resilience, the model performs input normalization to scale voltage, current, and temperature sequences into a stable range [ 0, 1].

**Figure 10:**
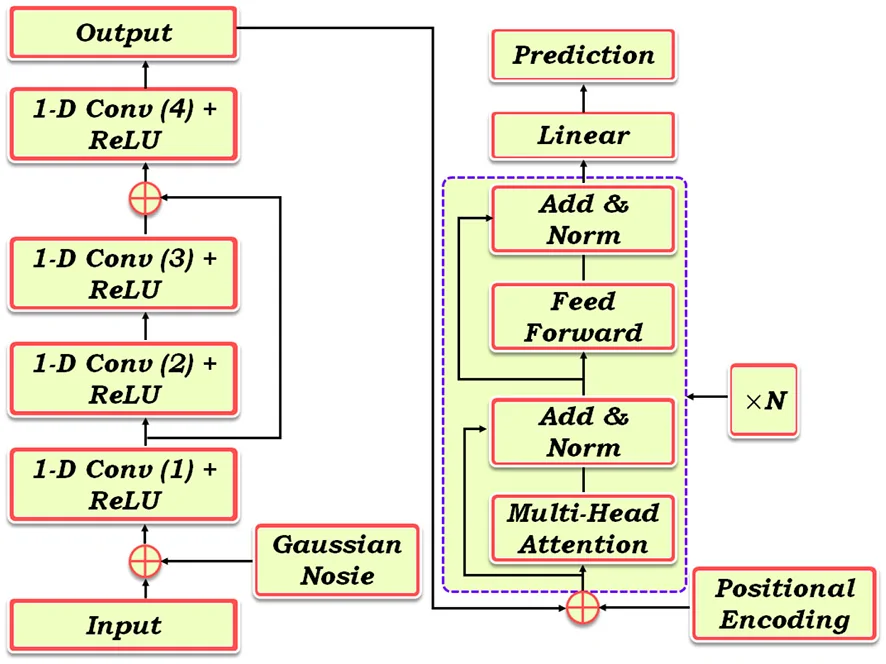
Denoising transformer-based neural network (DTNN)-based remaining useful life (RUL) prediction flowchart.

A 1-D convolutional denoising front-end with residual learning is used to minimize sensor disturbances and operating noise, allowing for the extraction of local electrochemical dynamics while maintaining notable degradation signs. A multi-head self-attention mechanism in stacked transformer encoder layers receives these encoded features and uses them to capture non-linear dependencies between previous and current operating states as in Equation 17:


$$\begin{array}{l} Attention\left(Q,K,V\right)=softmax\left(\frac{QK^{T}}{\sqrt{d_{h}}}\right)V \end{array}$$ (17)

For accurate RUL tracking under dynamic load conditions, this allows DTNN to learn charging/discharging trends over extended periods of time. A feed-forward network is used to further improve the extracted global temporal features as in Equation 18:


$$\begin{array}{l} FFN\left(x\right)=ReLU\left(xW_{1}+b_{1}\right)W_{2}+b_{2} \end{array}$$ (18)

and ultimately mapped through a fully connected prediction layer to SOC output provided in Equation 19:


$$\begin{array}{l} SoC_{t}=f\left(X_{1:t}\right) \end{array}$$ (19)

where *X*_1:*t*_ represents the battery measurement sequence up to time *t*. The DTNN model provides excellent robustness and high estimation accuracy for real-time SOC monitoring in BMS by combining noise suppression, local feature extraction, and long-range temporal attention (Han et al., 2023).

### 5.3 Transformer-based neural network

Accurate RUL assessment is essential for LiB dependability, safety, and predictive maintenance. RUL is often defined as the number of cycles needed for a battery to reach the EOL threshold, which is usually 70%−80% of the initial capacity because battery degradation is strongly reflected in SOH progression. The course of SOH deterioration is represented as in Equation 20:


$$\begin{array}{l} {\hat{x}}_{t}=f^{\prime }\left(W^{\prime }z+b^{\prime }\right) \end{array}$$ (20)

where *W*′ and *b*′ represent the prediction layer's weight and bias matrices, *z* represents the hidden representation that was extracted from the final transformer encoder, and *f*(·) stands for the non-linear mapping function that is used to predict the battery's future capacity.

The general denoising-transformer learning goal is expressed as in Equation 21:


$$\begin{array}{l} L=\overset{n}{\underset{t=T+1}{\sum }}{\left(x_{t}-{\hat{x}}_{t}\right)}^{2}+\alpha \overset{n}{\underset{i=1}{\sum }}l\left({\tilde{x}}_{i}-{\hat{x}}_{i}\right)+\lambda \Omega \left(\Theta \right) \end{array}$$ (21)

The prediction loss for future battery capacity estimation is represented by the first term; the reconstruction loss of the denoising autoencoder is represented by the second term; the contribution of denoising and prediction tasks is controlled by α; the regularization coefficient is represented by λ, and the trainable network parameters are represented by (Θ).

For battery RUL prediction, conventional RNN, LSTM, and GRU-based DL techniques are frequently employed. Nevertheless, their sequential training mechanism frequently results in excessive computational complexity and subpar long-term dependency learning. Because of their superior long-range temporal feature extraction capabilities and parallel attention mechanism, transformer-based neural networks have emerged as a highly effective option to address this constraint, as shown in Figure 11.

**Figure 11:**
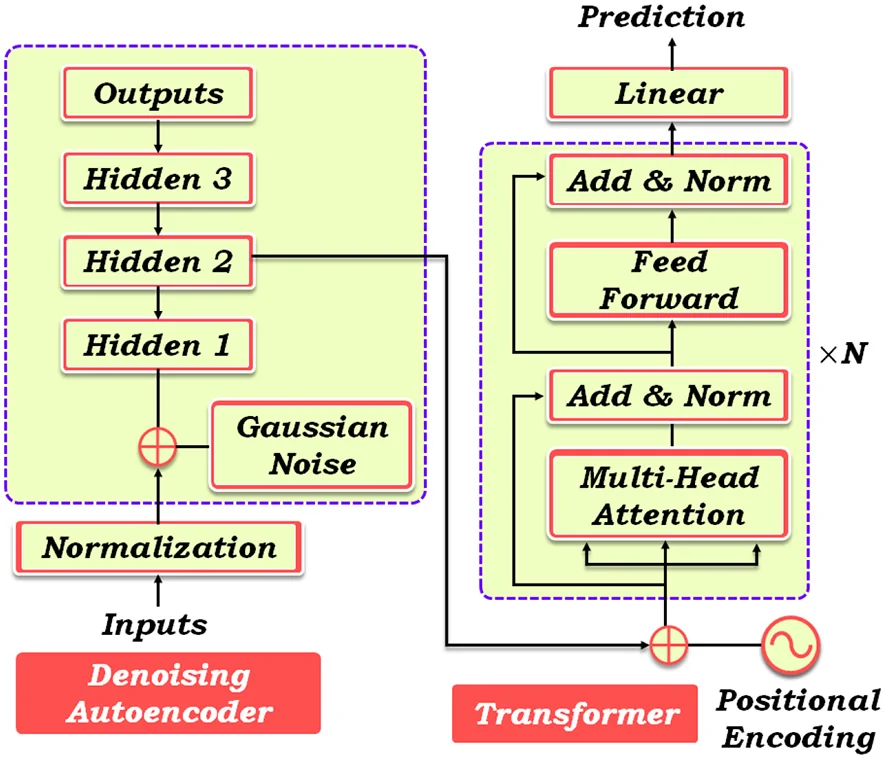
Remaining useful life (RUL) prediction using denoising transformer model.

The historical battery capacity sequence is first normalized in the transformer-based framework $\left(x^{\prime }=\frac{x}{C_{0}}\right)$ to reduce differences in cell distribution. A denoising autoencoder (DAE) is incorporated as a preprocessing block to reconstruct cleaner deterioration signals because raw battery records frequently contain regeneration noise $\left(z=\sigma \left(W\tilde{x}+b\right)\right)$ and measurement fluctuations $\left(\hat{x}=W^{\prime }z+b^{\prime }\;\right)$.

The transformer encoder is then used to process the denoised sequence; a multi-head self-attention mechanism captures deterioration relationships over distant cycles, and positional encoding preserves cycle-order information. This makes it possible for the model to learn long-term aging trends and local degradation fluctuations more efficiently than traditional recurrent designs. Finally, a fully linked prediction layer maps the battery's remaining cycle life to an estimate of future capacity deterioration (Chen D. et al., 2022).

### 5.4 Particle Swarm optimization (PSO)-based extreme learning machine (ELM)–relevance vector machine (RVM)

A PSO-based ELM–RVM framework is used to integrate fast non-linear learning, sparse probabilistic inference, and adaptive parameter optimization for precise RUL prediction. As shown in Figure 12, RVM improves the RUL prediction by mapping the extracted health-sensitive features into probabilistic RUL values with uncertainty bounds, while the ELM model first captures the non-linear temporal relationship between measurable battery signals such as voltage, current, temperature, and cycle count. In order to reduce estimation error and enhance convergence, the PSO algorithm is used to optimize the kernel hyperparameters of RVM and hidden-layer parameters of ELM.

**Figure 12:**
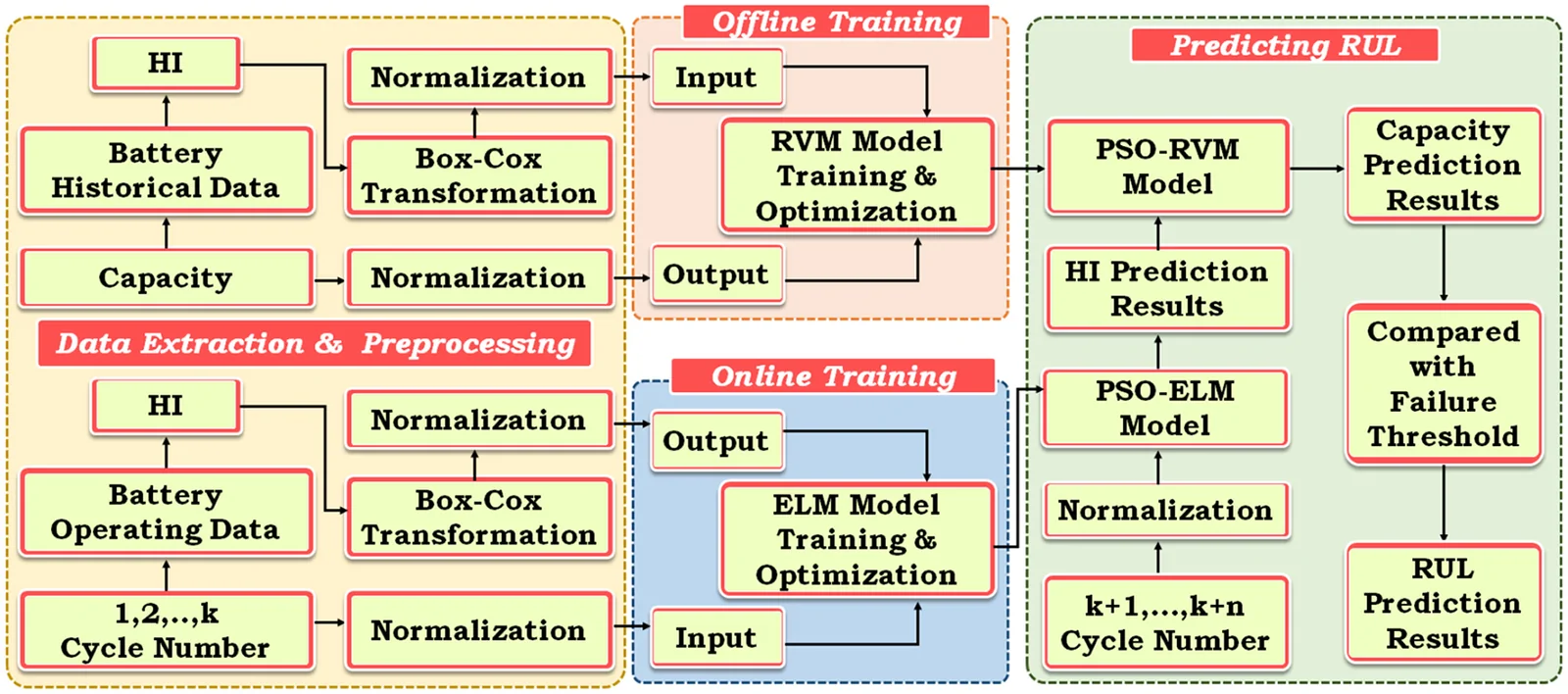
Particle swarm optimization–extreme learning machine–relevance vector machine (PSO-ELM-RVM) framework.

The mathematical representation of the ELM-based SOC predictor is provided in Equation 22:


$$\begin{array}{l} f\left(x\right)=\overset{L}{\underset{i=1}{\sum }}\beta_{i}g\left(w_{i}x+b_{i}\right) \end{array}$$ (22)

Here, *L* is the number of hidden neurons, *x* is the input feature vector made up of samples of current, terminal voltage, and temperature, and *g*(·) is the activation function, *w*_*i*_ and *b*_*i*_ are randomly initialized input weights and biases, and β_*i*_ represents the output weights that were calculated using the Moore-Penrose pseudoinverse as in Equation 23:


$$\begin{array}{l} \beta =H^{†}T \end{array}$$ (23)

where *T* is the target RUL vector and *H* is the hidden-layer output matrix. This hybrid PSO–ELM–RVM structure is ideal for EV BMS because it offers fast convergence, decreased overfitting, very accurate non-linear SOC tracking, and confidence-aware estimation (Yao F. et al., 2022). The joint battery-state estimating model, which integrates SOC, SOH, and RUL prediction frameworks for unified battery management, is the focus of the next section.

## 6 Joint battery-state estimation model

Recent research has focused on joint battery state estimation that allows for the simultaneous estimation of multiple health metrics (SOC, SOH, and RUL) for one battery in a single integrated model. This improves the consistency and reliability of estimates across all metrics and allows for shared feature usage among different metrics, which provides considerable benefits to BMS for EVs and grid storage. Table 3 summarizes recent research efforts in the field of joint battery state estimation by presenting data used for estimation, model types employed, target metrics, and reported estimation accuracy.

**Table 3:** Review of recent joint battery state estimation approaches for LiBs.

References	Cell type	Data type	Dataset	Estimation covered	Models used	Error
Zheng et al. (2025)	18650 Li-ion, Prismatic Li-ion	Voltage, capacity, cycle, noisy degradation features	NASA (B0005, B0006, B0007), CALCE (CS2–36, CS2–37, CS2–38)	SOH, RUL	ICEEMDAN + ODBN-BA (Optimal DBN with Bayesian Algorithm), compared with RBF, ELM, DBN, CNN-LSTM	SOH mean absolute error (MAE): 9.7261 × 10^−5^, RUL AE: 0 cycles
Zraibi et al. (2022)	18650 Li-ion	Voltage, capacity, cycle degradation data	NASA (B0005, B0006, B0007)	SOH, RUL	CNN-BGRU-DNN	SOH Root Mean Square Error (RMSE): 0.008 (B0006), 0.009 (B0007)
Nagarale and Patil (2023)	Panasonic 18650PF Li-ion	Voltage, current, temperature, drive-cycle data	NASA Panasonic 18650PF	SOC, SOH	LSTM, BiLSTM, FPGA-accelerated LSTM (PYNQ Z2)	SOC MAE: 0.016%, SOC RMSE: 0.454%, SOH MAE: 0.012%
Hu et al. (2023)	Lithium-ion battery pack (EV pack cells)	Voltage, current, charge/discharge cycle data, pack inconsistency features	Experimental EV battery pack testbed (700th−800th cycles)	SOC, SOH	NSSR-LSTM, LSTM-only, Ah-Integral	SOC error: 2.43% RMSE, SOH error: 1.3%
Wei et al. (2025)	Lithium-ion battery (automotive cells)	Voltage interval features, differential capacity, current–voltage product, temperature, cycle data	University of Michigan battery lab, McMaster University Hamilton	SOC, SOH	AR-ECM + MSIVY-HKELM + EKF + PSO	SOH error < 1%, SOC error < 2%, Avg. MAE: 0.6054%, RMSE: 0.5922%

From Table 3, the joint application of DL neural networks and hybrid intelligent optimization techniques consistently outperforms traditional methods. Amongst the techniques reviewed, the ICEEMDAN with ODBN-BA technique from (Zheng et al., 2025) has the best reported overall performance, with a reported MAE for SOH estimates of 9.7261 × 10^−5^ and an absolute error of zero cycles for remaining useful life estimates, making this method the best estimator of SOH/RUL. For joint SOC/SOH estimation, the results from (Nagarale and Patil, 2023) suggest that LSTM-based temporal learning algorithms provide excellent performance, supporting their use for multi-state battery estimation activities.

## 7 Comparative analysis of SOC estimation methods

This section examines the estimation accuracy, SOC tracking performance, and related error metrics across common drive-cycle datasets in order to provide a comparative analysis of SOC-based estimation models. The comparison focuses on root mean square error (RMSE), mean absolute error (MAE), and the coefficient of determination (*R*^2^), which together show resilience and the capacity for model generalization. The most dependable SOC estimator for real-time battery management applications is identified by comparing several DL and hybrid intelligence techniques.

SOC tracking approaches such as PSO-ASRCKF (Wang et al., 2024), TD-RNN (Xi et al., 2022), GA-GRU (Chen J. et al., 2022), and DA-GRU (Yang et al., 2022) using the same operational dataset are compared in Figure 13. While PSO-ASRCKF and GA-GRU show smoother convergence and reduced transient deviation, especially during rapid SOC dips, all algorithms closely track the reference SOC curve. Additionally, the DA-GRU model exhibits negligible steady-state volatility and extremely reliable tracking behavior. TD-RNN, on the other hand, has a greater deviation in the vicinity of acute transition regions, signifying a slower rate of adaptation under dynamic load fluctuations.

**Figure 13:**
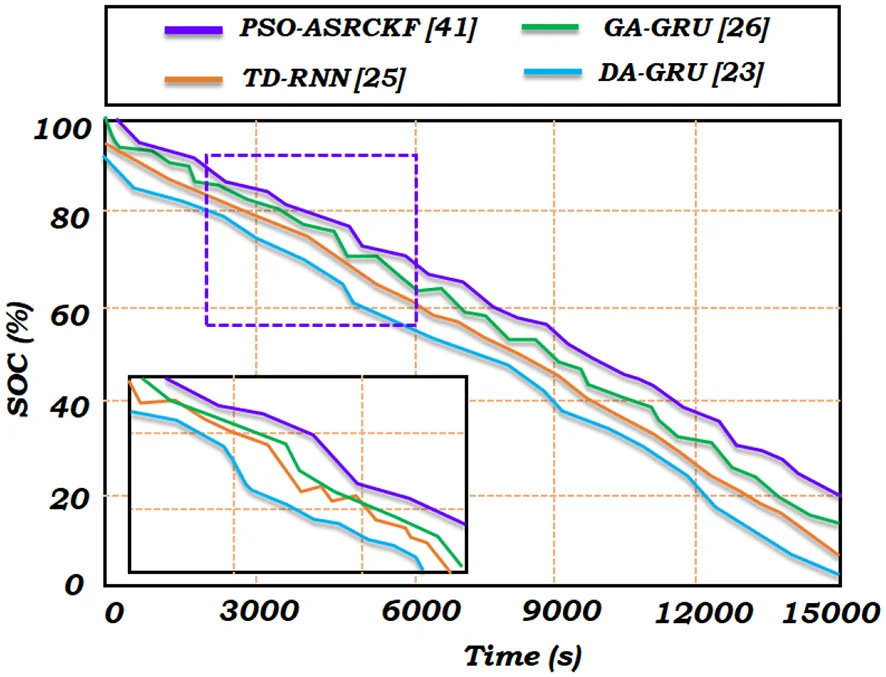
Comparative state-of-charge (SOC) estimation.

SOC estimation error variation between GRU-KF (Yang et al., 2023), DA-GRU (Yang et al., 2022), TD-RNN (Xi et al., 2022), GA-GRU (Chen J. et al., 2022), and PSO-ASRCKF (Wang et al., 2024) is presented in Figure 14. It shows that GA-GRU and PSO-ASRCKF maintain the narrowest error bands, indicating improved generalization and greater robustness. Additionally, DA-GRU achieves low dynamic error, especially under typical drive cycles. Compared to the optimized recurrent models, GRU-KF exhibits greater transient fluctuations than optimized neural network models.

**Figure 14:**
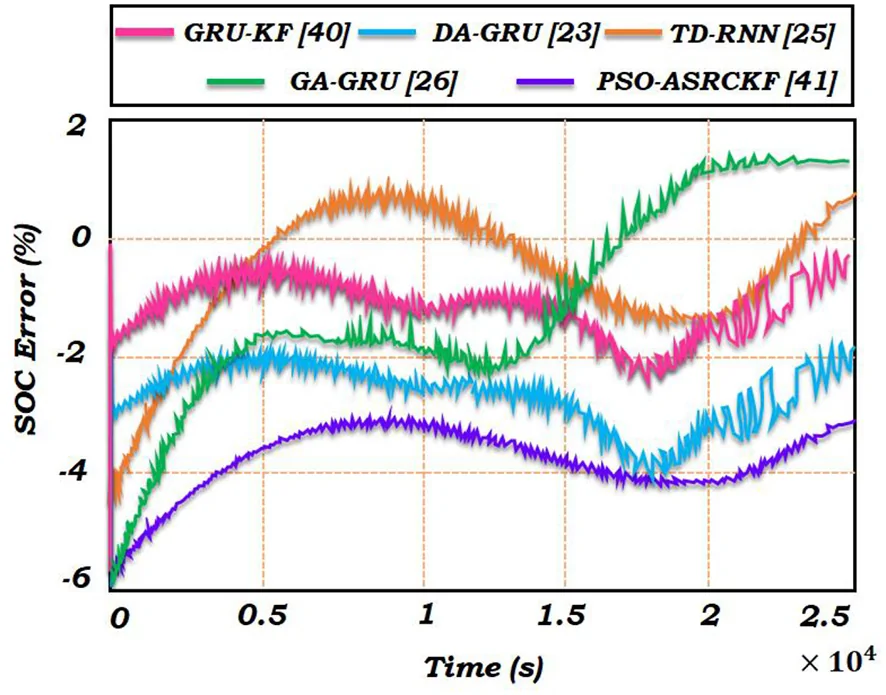
Error-distribution comparison of state-of-charge (SOC) estimation models.

A comparison of the MAE of several SOC estimation techniques is displayed in Figure 15, which demonstrates the superiority of deep recurrent and advanced hybrid models. PSO-ASRCKF (Wang et al., 2024) and Bi-LSTM (Zafar et al., 2024) collectively attain the lowest MAE of 0.0017, indicating the highest SOC forecast precision. CNN-BWGRU (Cui et al., 2022) with 0.00687 and ECM-EKF (Wang J. et al., 2023) with 0.0076, which both show outstanding estimation performance, come next. On the other hand, despite having good overall tracking performance, DA-GRU (Yang et al., 2022) records 0.3854, GRU-KF (Yang et al., 2023) 0.39, and GA-GRU (Xi et al., 2022) 0.1750, indicating comparatively higher absolute deviation.

**Figure 15:**
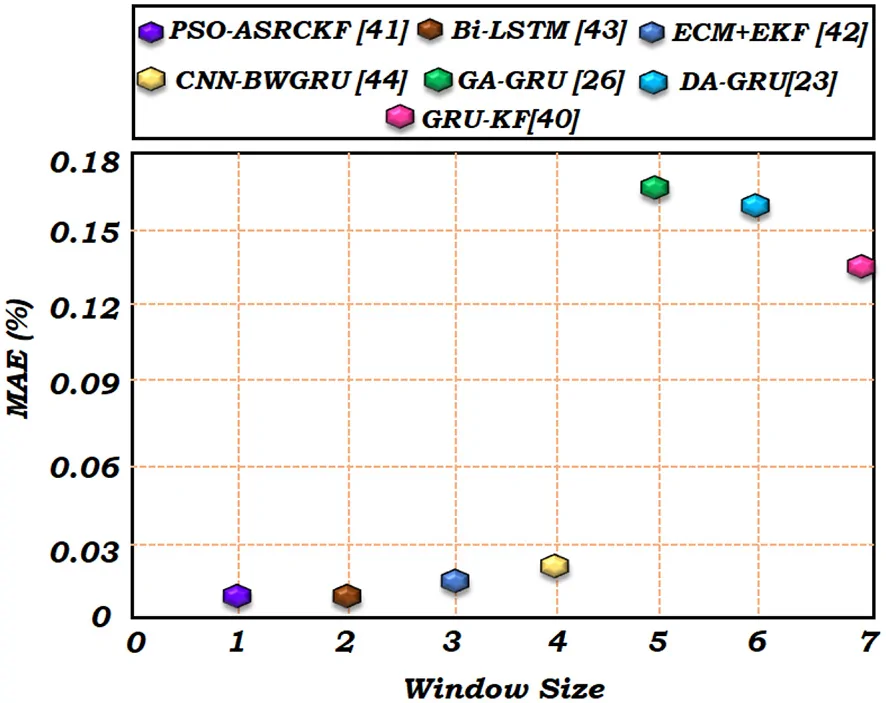
Mean absolute error (MAE)-based performance comparison.

The RMSE and *R*^2^ performance of SOC estimation models across popular drive-cycle datasets, such as USO6, FUDS, BJDST, HWFET, and UDDS, are compared in Table 4. With exceptionally low RMSE values of 0.00813, 0.00659, and 0.0022, as well as near-unity R2 values above 0.999, CNN-BWGRU (Cui et al., 2022) and Bi-LSTM (Zafar et al., 2024) outperform all other approaches in HWFET and UDDS cycles. Across the USO6, FUDS, and BJDST datasets, GA-GRU (Chen D. et al., 2022) consistently outperforms DA-GRU (Yang et al., 2022) in RMSE, proving the efficacy of optimization-assisted recurrent learning for reliable SOC estimation. Each model's RMSE and R2 values are taken directly from the corresponding reference articles. These metrics are not reported in the original papers for methods for which R2 values are not displayed.

**Table 4:** Comparative root mean square error (RMSE) and coefficient of determination (*R*^2^) analysis of SOC estimation models across standard drive-cycle datasets.

Model	RMSE	*R* ^2^
USO6 dataset		
DA-GRU (Yang et al., 2022)	0.4839	0.9997
GA-GRU (Chen J. et al., 2022)	0.0725	—
Bi-LSTM (Zafar et al., 2024)	6.42	0.9925
CNN-BWGRU (Cui et al., 2022)	0.10104	0.9986
FUDS dataset		
DA-GRU (Yang et al., 2022)	0.5131	0.9996
GA-GRU (Xi et al., 2022)	0.0851	—
BJDST dataset		
DA-GRU (Yang et al., 2022)	1.0435	0.9977
GA-GRU (Chen J. et al., 2022)	0.2295	—
HWFET dataset		
GRU-KF (Yang et al., 2023)	0.17	—
Bi-LSTM (Zafar et al., 2024)	0.0022	0.9935
CNN-BWGRU (Cui et al., 2022)	0.00813	0.9992
UDDS dataset		
GRU-KF (Yang et al., 2023)	0.47	—
ECM-EKF (Wang J. et al., 2023)	0.0076	—
Bi-LSTM (Zafar et al., 2024)	9.845	0.9905
CNN-BWGRU (Cui et al., 2022)	0.00659	0.9991

## 8 Comparative analysis of SOH estimation methods

The ability of SOH estimation models to track SOH degradation, the evolution of estimation errors, and related performance measures over battery cycles is compared in this section. The comparison highlights how various hybrid intelligent and DL models adhere to the non-linear capacity fading trend while minimizing estimation error. The most reliable model for long-term battery health prognosis is found by assessing both cycle-wise error behavior and SOH trajectory accuracy.

SOH deterioration trajectories predicted over 50 cycles by IPSO-CNN Bi-LSTM (Li D. et al., 2022), CNN-Transformer (Gu et al., 2023), GCN-MP (Zhou et al., 2024), ANN (Wei et al., 2022b), and CNN (Chen S. Z. et al., 2023) are compared in Figure 16. The general downward trend in battery health degradation is captured by all models. However, IPSO-CNN Bi-LSTM (Liang et al., 2025) most reliably tracks the degradation route, particularly during the sharp decline in SOH after 25 cycles. In the mid-cycle area, CNN-Transformer (Gu et al., 2023) overestimates SOH while maintaining comparatively smooth estimation. The tracking performance of GCN-MP (Zhou et al., 2024), ANN (Wei et al., 2022b), and CNN (Chen S. Z. et al., 2023) is more limited, with slight oscillations, signifying that they are less adaptive to non-linear aging changes.

**Figure 16:**
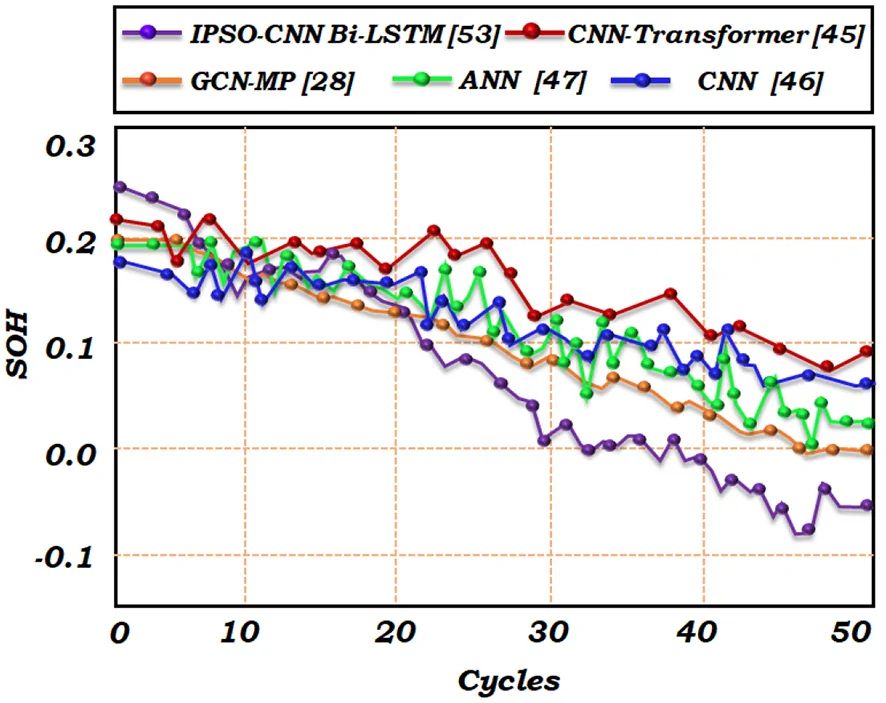
Comparative state-of-health (SOH) degradation tracking performance.

Cycle-wise SOH estimation error behavior of GCN-MP (Zhou et al., 2024), CNN-Transformer (Gu et al., 2023), and IPSO-CNN Bi-LSTM (Li D. et al., 2022) from 0 to 160 cycles is shown in Figure 17. The IPSO-CNN Bi-LSTM model's better robustness and generalization are confirmed by its lowest and most stable error range, which stays within 0–0.02 throughout the cycle range. Although it exhibits higher transient spikes around the 90th−110th cycles, CNN-Transformer (Gu et al., 2023) likewise performs well. GCN-MP (Zhou et al., 2024), on the other hand, records the largest fluctuation, with peak errors close to 0.14, suggesting greater susceptibility to noise and localized deterioration anomalies.

**Figure 17:**
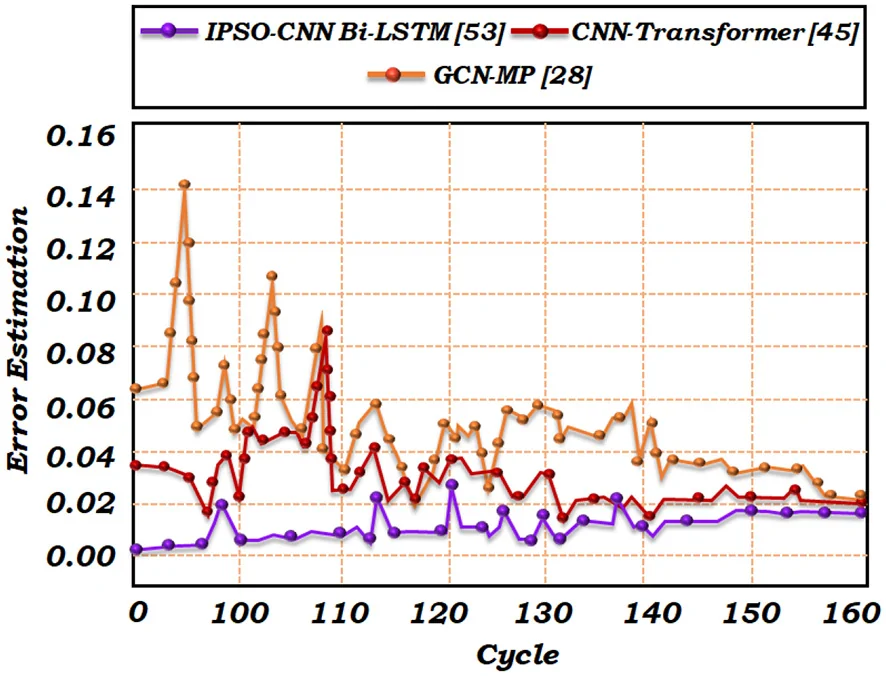
Cycle-wise state-of-health (SOH) estimation error comparison.

The Figure 18 comparison shows that different DL and hybrid intelligent models minimize estimation error while successfully following the non-linear capacity fading trend. ABC-BiGRU (Chen D. et al., 2022) outperforms *R*-ViT (Chen L. et al., 2023) (0.46), IALO-SVR (Li Q. et al., 2022) (0.62), ANN (Wei et al., 2022b) (0.85), and CNN (Chen S. Z. et al., 2023) (1.14) in the RMSE comparison, demonstrating its higher precision. *R*^2^ values show nearly perfect correlation for transformer and attention-based models, rising from QCNN (Liang et al., 2025) (0.9709) to IO-IMV-LSTM (Lyu et al., 2023) (0.99701), ABC-Bi-GRU (Chen D. et al., 2022) (0.9973), CNN-Transformer (Gu et al., 2023) (0.9987), and CNN-GRU-Att (Zhao et al., 2023) (0.9991). Based on the MAE results, CNN–Transformer (Gu et al., 2023) (0.0027) has the lowest absolute deviation, followed by LSTM (Mu et al., 2025) (0.0039), GCN–MP (Zhou et al., 2024) (0.0049), IO–IMV–LSTM (Lyu et al., 2023) (0.1849), and CNN–GRU-Att (Zhao et al., 2023) (0.322). The resilience of transformer-based models is further supported by the MAPE study, where CNN-Transformer (Gu et al., 2023) achieves the lowest percentage error (0.0037), outperforming ABC-Bi-GRU (Chen D. et al., 2022) (0.0130), IO-IMV-LSTM (Lyu et al., 2023) (0.2015), QCNN (Liang et al., 2025) (0.55), and IALO-SVR (Li Q. et al., 2022) (0.79). Overall, the reported results indicate that transformer-integrated and attention-enhanced hybrid models are the most dependable options for long-term battery health prognosis since they best capture the non-linear SOH trajectory and reduce cycle-wise estimation errors.

**Figure 18:**
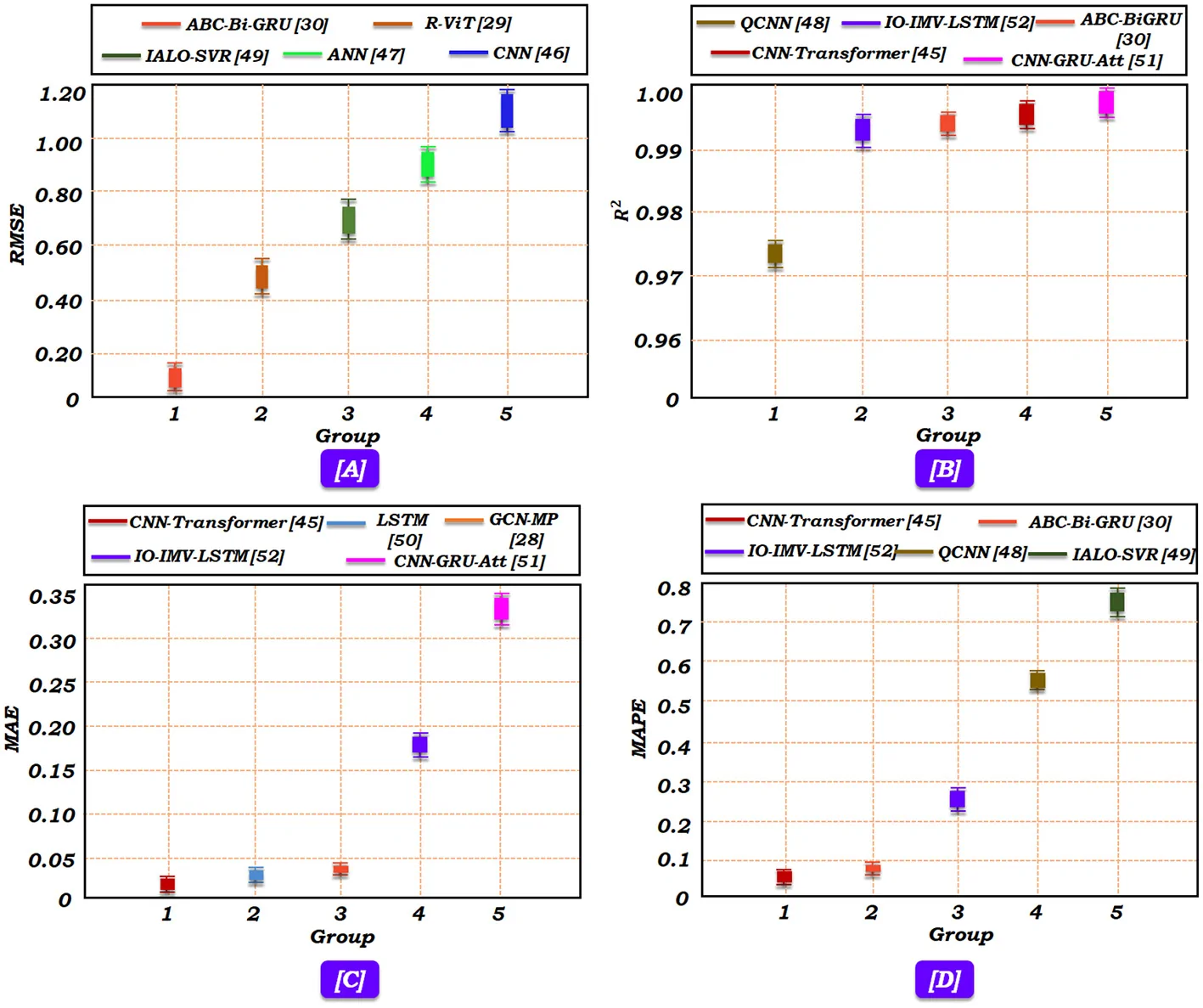
Comparative evaluation of remaining useful life (RUL) prediction. **(A)** RMSE **(B)** R2 **(C)** MAE **(D)** MAPE.

## 9 Comparative analysis of RUL estimation methods

RUL forecast accuracy, cycle-wise prediction error, and standard error metrics of RUL-based estimation models are compared across benchmark battery datasets in this section. In order to evaluate prediction reliability, the comparison focuses on Relative Error (RE), MAE, RMSE, coefficient of determination (*R*^2^), and mean absolute percentage error (MAPE). The most reliable data-driven models for long-term battery failure prognosis and maintenance scheduling are identified using these metrics.

RUL prediction performance of the current DL and hybrid models using the NASA and CALCE datasets is presented in Table 5. CS-VMD-GRU (Ding et al., 2022) has the lowest MAE (0.0114) and RMSE (0.0142) for the NASA dataset, indicating extremely precise failure cycle prediction. MMMe (Zhao L. et al., 2024) records the lowest RE of 0.0019 for the CALCE dataset, while CS-VMD-GRU (Ding et al., 2022) once more displays the best MAE and RMSE values, indicating robust cross-dataset generalization. In long-horizon battery life predictions, hybrid decomposition-assisted recurrent models often perform better than transformer-only designs.

**Table 5:** Comparative relative error (RE), mean absolute error (MAE), and root mean square error (RMSE) metrics of RUL estimation models on NASA and CALCE datasets.

Model	RE	MAE	RMSE
NASA dataset			
AttMoE (Chen and Zhou, 2024)	0.2000	0.0760	0.0872
CA-LSTM (Chen C. et al., 2023)	0.0084	0.0179	0.0251
CS-VMD-GRU (Ding et al., 2022)	—	0.0114	0.0142
MMMe (Zhao L. et al., 2024)	0.005	0.04	0.0515
CALCE dataset			
AttMoE (Chen and Zhou, 2024)	0.0761	0.0577	0.0794
CA-LSTM (Chen C. et al., 2023)	0.0231	0.0309	0.0401
CS-VMD-GRU (Ding et al., 2022)	—	0.0204	0.0207
MMMe (Zhao L. et al., 2024)	0.0019	0.229	0.0306

Table 6 displays an *R*^2^-based comparison of goodness-of-fit for RUL estimation techniques. With an improved *R*^2^ value of 0.9954, the CNN Bi-LSTM Attention model (Zhao F. M. et al., 2024) shows the strongest association between projected and real battery end-of-life trajectories. With *R*^2^ values above 0.99, DTNN (Han et al., 2023) and Conv 1-D SAM (Li et al., 2023) also provide superior fitting capacity, demonstrating the efficacy of temporal convolutional and attention-enhanced recurrent learning frameworks.

**Table 6:** Coefficient of determination (*R*^2^) comparison of RUL estimation models.

Model	*R* ^2^
CNN Bi-LSTM Att (Zhao F. M. et al., 2024)	0.9954
Conv 1-D SAM (Li et al., 2023)	0.99
DTNN (Han et al., 2023)	0.991
DE-GWO LSTM (Wang S. et al., 2023)	0.9426
AccuCell prodigy (Iftikhar et al., 2024)	0.9849

The MAPE of RUL estimation techniques is demonstrated in Table 7. The most accurate percentage-based RUL prediction is obtained by CNN Bi-LSTM Att (Zhao F. M. et al., 2024), which has the lowest MAPE of 0.0056 out of all the techniques. While CPSO-PF (Ye et al., 2023) exhibits a comparatively higher percentage deviation, DE-GWO LSTM (Wang S. et al., 2023) and DTNN (Han et al., 2023) both show competitive performance.

**Table 7:** Mean absolute percentage error (MAPE) comparison of RUL estimation models.

Model	MAPE
CNN Bi-LSTM Att (Zhao F. M. et al., 2024)	0.5481
DTNN (Han et al., 2023)	0.632
DE-GWO LSTM (Wang S. et al., 2023)	1.1478
CPSO-PF (Ye et al., 2023)	3.0056

### 9.1 Critical comparison of AI-based methods

This section provides a thorough analysis of AI-based battery prognostic models by comparing the estimation of SOC, SOH, and RUL across different datasets. While CNN-BWGRU, Bi-LSTM, and CS-VMD-GRU DL architectures demonstrate high classification accuracy and good generalizability, their relative performance varies depending on dataset complexity and the operational dynamics of each test. In addition, recurrent models that use optimization methods converge more smoothly and have a significantly smaller transient deviation when compared to transformer-based architectures, which have the highest correlation coefficients and demonstrate the most robust behavior in non-linear degradation cases.

## 10 Discussion

There are still challenges in AI-based battery prognostics. Model explainability is very limited, making it difficult to interpret model decision-making in safety-critical BMS environments. The inability to generate enough data and a shift between laboratory conditions and real-world operations restrict model transferability. There is also a need for ongoing research to address robustness issues associated with noise applied to voltage and current measurements, as well as to quantify uncertainty associated with predictions. Therefore, it is critical to address these issues by integrating physics-informed hybridization, transfer learning, and explainable AI (XAI) into battery health management frameworks to create interpretable, reliable, and computationally efficient models suitable for edge deployment.

## 11 Conclusion

In this analysis of AI-driven lithium-ion battery state estimation tasks, DL and hybrid intelligent optimization models consistently demonstrate strong predictive performance and robustness. The CNN-BWGRU (Wang et al., 2024) and Bi-LSTM (Yang et al., 2023) models perform best overall for SOC prediction, reporting exceptionally low error values across several drive-cycle datasets, especially RMSE values in the range of 0.00659–0.00813 and MAE as low as 0.0017, which validates their exceptional ability to track dynamic charge variations under realistic operating conditions. The most successful method for SOH estimation is IPSO-CNN Bi-LSTM (Liang et al., 2025), which provides significant robustness against non-linear aging patterns and noisy degradation data, as well as extremely steady degradation trajectory tracking with the lowest cycle-wise error band of roughly 0–0.02. CS-VMD-GRU (Lyu et al., 2023) and CPSO-PF (Wang S. et al., 2023) approaches provide the best results for RUL prediction: CS-VMD-GRU achieves the lowest MAE and RMSE across both NASA and CALCE datasets, while CPSO-PF records the lowest MAPE of 0.0056, indicating highly accurate end-of-life forecasting. Overall, the review demonstrates that the most promising path for next-generation BMS is hybrid DL architectures combined with swarm optimization, Bayesian learning, and attention mechanisms. Of the three estimation tasks, decomposition-assisted recurrent learning is most effective for RUL prediction, optimization-enhanced CNN–Bi-LSTM frameworks are most effective for SOH estimation, and bidirectional recurrent convolutional models yield the highest precision for SOC estimation. Future lithium-ion battery health management systems should therefore increasingly use multi-feature hybrid AI frameworks that jointly estimate SOC, SOH, and RUL with high robustness, low uncertainty, and strong real-time adaptability. This makes them ideal for advanced energy storage systems, smart grids, and EVs.

### 11.1 Future directions

- Explainable AI (XAI): addresses interpretability and confidence in AI-based diagnoses.
- Hybrid physics-based and data-driven integration: creates cohesive frameworks that integrate intelligent learning and electrochemical modeling.
- Cross-domain generalization and transfer learning: enables strong adaptation across chemistries, datasets, and operating conditions.
- Edge-deployment constraints: maximizing computational efficiency for embedded real-time applications.

By taking these steps, AI-enabled battery prognostics will become more dependable, transparent, and scalable, opening the door to safer, more durable, and sustainable ESSs.

## References

[B1] Amir S. Gulzar M. Tarar M. O. Naqvi I. H. Zaffar N. A. Pecht M. G. (2022). Dynamic equivalent circuit model to estimate state-of-health of lithium-ion batteries. IEEE Access. 10, 18279–18288. doi: 10.1109/ACCESS.2022.3148528

[B2] Che Y. Zheng Y. Wu Y. Lin X. Li J. Hu X. . (2023). Battery states monitoring for electric vehicles based on transferred multi-task learning. IEEE Transac. Vehicul. Technol. 72, 10037–10047. doi: 10.1109/TVT.2023.3260466

[B3] Chen C. Wei J. Li Z. (2023). Remaining useful life prediction for lithium-ion batteries based on a hybrid deep learning model. Processes. 11:2333. doi: 10.3390/pr11082333

[B4] Chen D. Weicong H. Xiuze Z. (2022). Transformer network for remaining useful life prediction of lithium-ion batteries. IEEE Access. 10, 19621–19628. doi: 10.1109/ACCESS.2022.3151975

[B5] Chen D. Zhou X. (2024). AttMoE: Attention with Mixture of Experts for remaining useful life prediction of lithium-ion batteries. J. Energy Storage. 84:110780. doi: 10.1016/j.est.2024.110780

[B6] Chen J. Lu C. Chen C. Cheng H. Xuan D. (2022). An improved gated recurrent unit neural network for state-of-charge estimation of lithium-ion battery. Appl. Sci. 12:2305. doi: 10.3390/app12052305

[B7] Chen L. Xie S. Lopes A. M. Bao X. (2023). A vision transformer-based deep neural network for state of health estimation of lithium-ion batteries. Internat. J. Elec. Power Energy Syst. 152:109233. doi: 10.1016/j.ijepes.2023.109233

[B8] Chen S. Z. Liang Z. Yuan H. Yang L. Xu F. Fan Y. (2023). A novel state of health estimation method for lithium-ion batteries based on constant-voltage charging partial data and convolutional neural network. Energy. 283:129103. doi: 10.1016/j.energy.2023.129103

[B9] Cui Z. Kang L. Li L. Wang L. Wang K. (2022). A hybrid neural network model with improved input for state of charge estimation of lithium-ion battery at low temperatures. Renewable Energy. 198, 1328–1340. doi: 10.1016/j.renene.2022.08.123

[B10] Deng Y. Du C. Ren Z. (2025). A novel method for estimating the state of health of lithium-ion batteries based on physics-informed neural network. Batteries. 11:49. doi: 10.3390/batteries11020049

[B11] Ding G. Wang W. Zhu T. (2022). Remaining useful life prediction for lithium-ion batteries based on CS-VMD and GRU. IEEE Access. 10, 89402–89413. doi: 10.1109/ACCESS.2022.3167759

[B12] Fu L. Jiang B. Zhu J. Wei X. Dai H. (2025). Early remaining useful life prediction for lithium-ion batteries using a gaussian process regression model based on degradation pattern recognition. Batteries. 11:221. doi: 10.3390/batteries11060221

[B13] Gomathi S. (2025). “Predictive intelligence for battery state-of-health: a machine learning approach to energy reliability,” in 2025 10th international conference on communication and electronics systems (ICCES). (Coimbatore: IEEE), 419–425. doi: 10.1109/ICCES67310.2025.11336954

[B14] Gu X. See K. W. Li P. Shan K. Wang Y. Zhao L. Lim K. C. Zhang N. (2023). A novel state-of-health estimation for the lithium-ion battery using a convolutional neural network and transformer model. Energy. 262:125501. doi: 10.1016/j.energy.2022.125501

[B15] Guo R. Shen W. (2022). Lithium-ion battery state of charge and state of power estimation based on a partial-adaptive fractional-order model in electric vehicles. IEEE Transac. Industrial Elec. 70, 10123–10133. doi: 10.1109/TIE.2022.3220881

[B16] Han Y. Li C. Zheng L. Lei G. Li L. (2023). Remaining useful life prediction of lithium-ion batteries by using a denoising transformer-based neural network. Energies. 16:6328. doi: 10.3390/en16176328

[B17] Hu P. Tang W. F. Li C. H. Mak S. L. Li C. Y. Lee C. C. (2023). Joint state of charge (SOC) and state of health (SOH) estimation for lithium-ion batteries packs of electric vehicles based on NSSR-LSTM neural network. Energies. 16:5313. doi: 10.3390/en16145313

[B18] Iftikhar M. Shoaib M. Altaf A. Iqbal F. Villar S. G. Dzul López L. A. . (2024). A deep learning approach to optimize remaining useful life prediction for Li-ion batteries. Sci. Rep. 14:25838. doi: 10.1038/s41598-024-77427-1 39468251 PMC11519505

[B19] Jafari S. Byun Y. C. (2022). Prediction of the battery state using the digital twin framework based on the battery management system. IEEE Access. 10, 124685–124696. doi: 10.1109/ACCESS.2022.3225093

[B20] Jafari S. Byun Y. C. (2023). Optimizing battery RUL prediction of lithium-ion batteries based on Harris hawk optimization approach using random forest and LightGBM. IEEE Access. 11, 87034–87046. doi: 10.1109/ACCESS.2023.3304699

[B21] Karthick A. (2025). Review on state of charge prediction of battery management system in electric vehicles. Proc. Institution Mech. Eng. Part D: J. Automob. Eng. 240:9. doi: 10.1177/09544070251380931

[B22] Karthick A. Yamuna M. Kaliappan S. Sakthi T. (2025). Artificial neural network-based SOC estimation under variable load conditions. 2025 11th international conference on electrical energy systems (Chennai: ICEES), 868–871. doi: 10.1109/ICEES67011.2025.11213289

[B23] Knox J. Blyth M. Hales A. (2024). Advancing state estimation for lithium-ion batteries with hysteresis through systematic extended Kalman filter tuning. Sci. Rep. 14:12472. doi: 10.1038/s41598-024-61596-0 38816427 PMC11139915

[B24] Li D. Yang D. Li L. Wang L. Wang K. (2022). Electrochemical impedance spectroscopy based on the state of health estimation for lithium-ion batteries. Energies. 15:6665. doi: 10.3390/en15186665

[B25] Li J. Zhao S. Miah M. S. Niu M. (2023). Remaining useful life prediction of lithium-ion batteries via an EIS based deep learning approach. Energy Rep.. 10, 3629–3638. doi: 10.1016/j.egyr.2023.10.030

[B26] Li Q. Li D. Zhao K. Wang L. Wang K. (2022). State of health estimation of lithium-ion battery based on improved ant lion optimization and support vector regression. J. Energy Storage. 50:104215. doi: 10.1016/j.est.2022.104215

[B27] Li Y. Li K. Liu X. Li X. Zhang L. Rente B. Sun T. Grattan K. T. (2022). A hybrid machine learning framework for joint SOC and SOH estimation of lithium-ion batteries assisted with fiber sensor measurements. Appl. Energy. 325:119787. doi: 10.1016/j.apenergy.2022.119787

[B28] Liang C. Tao S. Huang X. Wang Y. Xia B. Zhang X. (2025). Stochastic state of health estimation for lithium-ion batteries with automated feature fusion using quantum convolutional neural network. J. Energy Chem. 106, 205–219. doi: 10.1016/j.jechem.2025.02.030

[B29] Lu J. Xiong R. Tian J. Wang C. Sun F. (2023). Deep learning to estimate lithium-ion battery state of health without additional degradation experiments. Nature Communications. 14:2760. doi: 10.1038/s41467-023-38458-w 37179411 PMC10183024

[B30] Lyu G. Zhang H. Miao Q. (2023). An interpretable state of health estimation method for lithium-ion batteries based on multi-category and multi-stage features. Energy. 283:129067. doi: 10.1016/j.energy.2023.129067

[B31] Mu G. Wei Q. Xu Y. Li J. Zhang H. Yang F. . (2025). State of health estimation of lithium-ion batteries based on feature optimization and data-driven models. Energy. 316:134578. doi: 10.1016/j.energy.2025.134578

[B32] Nagarale S. D. Patil B. P. (2023). Accelerating AI-Based Battery Management System's SOC and SOH on FPGA. Appl. Computat. Intelligence Soft Comput. 2023:2060808. doi: 10.1155/2023/2060808

[B33] Natella D. Onori S. Vasca F. (2022). A co-estimation framework for state of charge and parameters of lithium-ion battery with robustness to aging and usage conditions. IEEE Transac. Industrial Electronics. 70, 5760–5770. doi: 10.1109/TIE.2022.3194576

[B34] Peng J. Meng J. Du X. Cai L. Stroe D. I. (2022). A fast impedance measurement method for lithium-ion battery using power spectrum property. IEEE Transac. Industrial Informat. 19, 8253–8261. doi: 10.1109/TII.2022.3217474

[B35] Pramila V. Rudhra S. Vinod S. Lakshmi D. (2025). Enhancing demand response and energy management in multi-microgrid systems with renewable energy sources. Renewable Energy. 253:123490. doi: 10.1016/j.renene.2025.123490

[B36] Qin Y. Arunan A. Yuen C. (2023). Digital twin for real-time Li-ion battery state of health estimation with partially discharged cycling data. IEEE Transac. Industrial Informat. 19, 7247–7257. doi: 10.1109/TII.2022.3230698

[B37] Qin Y. Yuen C. Yin X. Huang B. (2022). A transferable multistage model with cycling discrepancy learning for lithium-ion battery state of health estimation. IEEE Transac. Industrial Informat. 19, 1933–1946. doi: 10.1109/TII.2022.3205942

[B38] Shu X. Chen Z. Shen J. Guo F. Zhang Y. Liu Y. (2022). State of charge estimation for lithium-ion battery based on hybrid compensation modeling and adaptive H-infinity filter. IEEE Transac. Transp. Electrific. 9, 945–957. doi: 10.1109/TTE.2022.3180077

[B39] Singh S. Ebongue Y. E. Rezaei S. Birke K. P. (2023). Hybrid modeling of lithium-ion battery: Physics-informed neural network for battery state estimation. Batteries. 9:301. doi: 10.3390/batteries9060301

[B40] Surya S. Samanta A. Marcis V. Williamson S. (2022). Hybrid electrical circuit model and deep learning-based core temperature estimation of lithium-ion battery cell. IEEE Transac. Transportation Electrific.. 8, 3816–3824. doi: 10.1109/TTE.2022.3170359

[B41] Wang J. Meng J. Peng Q. Liu T. Zeng X. Chen G. Li Y. (2023). Lithium-ion battery state-of-charge estimation using electrochemical model with sensitive parameters adjustment. Batteries. 9:180. doi: 10.3390/batteries9030180

[B42] Wang S. Fan Y. Jin S. Takyi-Aninakwa P. Fernandez C. (2023). Improved anti-noise adaptive long short-term memory neural network modeling for the robust remaining useful life prediction of lithium-ion batteries. Reliability Eng. Syst. Safety. 230:108920. doi: 10.1016/j.ress.2022.108920

[B43] Wang S. Zhang S. Wen S. Fernandez C. (2024). An accurate state-of-charge estimation of lithium-ion batteries based on improved particle swarm optimization-adaptive square root cubature kalman filter. J. Power Sources. 624:235594. doi: 10.1016/j.jpowsour.2024.235594

[B44] Wang Y. Han X. Guo D. Lu L. Chen Y. Ouyang M. (2022). Physics-informed recurrent neural network with fractional-order gradients for state-of-charge estimation of lithium-ion battery. IEEE J. Radio Freq. Identification. 6, 968–971. doi: 10.1109/JRFID.2022.3211841

[B45] Wang Z. Feng G. Liu X. Gu F. Ball A. (2022). A novel method of parameter identification and state of charge estimation for lithium-ion battery energy storage system. J. Energy Storage. 49:104124. doi: 10.1016/j.est.2022.104124

[B46] Wei Z. Hu J. He H. Yu Y. Marco J. (2022a). Embedded distributed temperature sensing enabled multistate joint observation of smart lithium-ion battery. IEEE Transac. Industrial Electronics. 70, 555–565. doi: 10.1109/TIE.2022.3146503

[B47] Wei Z. Ruan H. Li Y. Li J. Zhang C. He H. (2022b). Multistage state of health estimation of lithium-ion battery with high tolerance to heavily partial charging. IEEE Transac. Power Electronics. 37, 7432–7442. doi: 10.1109/TPEL.2022.3144504

[B48] Wei Z. Sun X. Li Y. Liu W. Liu C. Lu H. (2025). A joint estimation method for the soc and soh of lithium-ion batteries based on ar-ecm and data-driven model fusion. Electronics. 14:1290. doi: 10.3390/electronics14071290

[B49] Xi Z. Wang R. Fu Y. Mi C. (2022). Accurate and reliable state of charge estimation of lithium ion batteries using time-delayed recurrent neural networks through the identification of overexcited neurons. Appl. Energy. 305:117962. doi: 10.1016/j.apenergy.2021.117962

[B50] Yang K. Tang Y. Zhang S. Zhang Z. (2022). A deep learning approach to state of charge estimation of lithium-ion batteries based on dual-stage attention mechanism. Energy. 244:123233. doi: 10.1016/j.energy.2022.123233

[B51] Yang Y. Zhao L. Yu Q. Liu S. Zhou G. Shen W. (2023). State of charge estimation for lithium-ion batteries based on cross-domain transfer learning with feedback mechanism. J. Energy Storage. 70:108037. doi: 10.1016/j.est.2023.108037

[B52] Yao F. He W. Wu Y. Ding F. Meng D. (2022). Remaining useful life prediction of lithium-ion batteries using a hybrid model. Energy. 248:123622. doi: 10.1016/j.energy.2022.123622

[B53] Yao Q. Lu D. D. C. Lei G. (2022). A surface temperature estimation method for lithium-ion battery using enhanced GRU-RNN. IEEE Transac. Transp. Electrif. 9, 1103–1112. doi: 10.1109/TTE.2022.3197927

[B54] Ye L. H. Chen S. J. Shi Y. F. Peng D. H. Shi A. P. (2023). Remaining useful life prediction of lithium-ion battery based on chaotic particle swarm optimization and particle filter. Internat. J. Electrochem. Sci. 18:100122. doi: 10.1016/j.ijoes.2023.100122

[B55] Zafar M. H. Khan N. M. Abou Houran M. Mansoor M. Akhtar N. Sanfilippo F. (2024). A novel hybrid deep learning model for accurate state of charge estimation of Li-Ion batteries for electric vehicles under high and low temperature. Energy. 292:130584. doi: 10.1016/j.energy.2024.130584

[B56] Zhao F. M. Gao D. X. Cheng Y. M. Yang Q. (2024). Application of state of health estimation and remaining useful life prediction for lithium-ion batteries based on AT-CNN-BiLSTM. Sci. Rep. 14:29026. doi: 10.1038/s41598-024-80421-2 39578515 PMC11584646

[B57] Zhao H. Chen Z. Shu X. Shen J. Lei Z. Zhang Y. (2023). State of health estimation for lithium-ion batteries based on hybrid attention and deep learning. Reliability Eng. Syst. Safety. 232:109066. doi: 10.1016/j.ress.2022.109066

[B58] Zhao L. Song S. Wang P. Wang C. Wang J. Guo M. (2024). A MLP-Mixer and mixture of expert model for remaining useful life prediction of lithium-ion batteries. Front. Computer Sci. 18:185329. doi: 10.1007/s11704-023-3277-4

[B59] Zheng R. Yang B. Qian Y. Li H. Gao D. Jiang L. (2025). Joint SOH and RUL estimation for lithium-ion batteries via optimal deep belief network with Bayesian algorithm. J. Energy Storage. 114:115891. doi: 10.1016/j.est.2025.115891

[B60] Zhou K. Q. Qin Y. Yuen C. (2024). Graph neural network-based lithium-ion battery state of health estimation using partial discharging curve. J. Energy Storage. 100:113502. doi: 10.1016/j.est.2024.113502

[B61] Zraibi B. Mansouri M. Loukili S. E. Alla S. B. (2022). Hybrid neural network method for predicting the soh and rul of lithium-ion batteries. Adv Sci. Technol. Eng. Syst. J. 7, 193–198. doi: 10.25046/aj070520

